# Calcium Entry through TRPV1: A Potential Target for the Regulation of Proliferation and Apoptosis in Cancerous and Healthy Cells

**DOI:** 10.3390/ijms21114177

**Published:** 2020-06-11

**Authors:** Kevin Zhai, Alena Liskova, Peter Kubatka, Dietrich Büsselberg

**Affiliations:** 1 Department of Physiology and Biophysics, Weill Cornell Medicine-Qatar, Education City, Qatar Foundation, Doha, PO Box 24144, Qatar; kez4003@qatar-med.cornell.edu; 2 Clinic of Obstetrics and Gynecology, Jessenius Faculty of Medicine, Comenius University in Bratislava, 03601 Martin, Slovakia; alenka.liskova@gmail.com; 3 Department of Medical Biology, Jessenius Faculty of Medicine, Comenius University in Bratislava, 03601 Martin, Slovakia; peter.kubatka@uniba.sk

**Keywords:** TRPV1, calcium signaling, apoptosis, proliferation, capsaicin, capsazepine, cancers

## Abstract

Intracellular calcium (Ca^2+^) concentration ([Ca^2+^]_i_) is a key determinant of cell fate and is implicated in carcinogenesis. Membrane ion channels are structures through which ions enter or exit the cell, depending on the driving forces. The opening of transient receptor potential vanilloid 1 (TRPV1) ligand-gated ion channels facilitates transmembrane Ca^2+^ and Na^+^ entry, which modifies the delicate balance between apoptotic and proliferative signaling pathways. Proliferation is upregulated through two mechanisms: (1) ATP binding to the G-protein-coupled receptor P2Y2, commencing a kinase signaling cascade that activates the serine-threonine kinase Akt, and (2) the transactivation of the epidermal growth factor receptor (EGFR), leading to a series of protein signals that activate the extracellular signal-regulated kinases (ERK) 1/2. The TRPV1-apoptosis pathway involves Ca^2+^ influx and efflux between the cytosol, mitochondria, and endoplasmic reticulum (ER), the release of apoptosis-inducing factor (AIF) and cytochrome c from the mitochondria, caspase activation, and DNA fragmentation and condensation. While proliferative mechanisms are typically upregulated in cancerous tissues, shifting the balance to favor apoptosis could support anti-cancer therapies. TRPV1, through [Ca^2+^]_i_ signaling, influences cancer cell fate; therefore, the modulation of the TRPV1-enforced proliferation–apoptosis balance is a promising avenue in developing anti-cancer therapies and overcoming cancer drug resistance. As such, this review characterizes and evaluates the role of TRPV1 in cell death and survival, in the interest of identifying mechanistic targets for drug discovery.

## 1. [Ca^2+^]_i_ and the Critical Balance between Apoptosis and Proliferation

Molecular mechanisms that mediate cell death and proliferation exist in balance in functional physiological systems. Proliferation is involved in structural development and renewal, while programmed cell death is necessary to eliminate defective cells and prevent uncontrolled growth. Carcinogenesis results from imbalances in the described pathways, which favor proliferation and reduce apoptosis [1,2]. Therefore, anti-cancer therapies shift the balance in the opposite direction by reducing proliferation and upregulating apoptosis.

Apoptosis is defined as programmed cell death, characterized by fragmentation of inter-nucleosomal DNA [3]. Two major mechanisms of apoptosis are an extrinsic, death-receptor mediated mechanism, and an intrinsic, mitochondria-mediated mechanism [4]. The extrinsic mechanism involves the linking of membrane death receptors to adapter proteins, which bind and position pro-caspase 8 for conversion into caspase 8; the intrinsic mechanism is triggered by the release of cytochrome *c* from mitochondria, which promotes caspase 9 activation [4,5]. The Bcl-2 family of proteins, which includes the proapoptotic proteins Bax and Bak and the antiapoptotic protein Bcl-2, is implicated in the intrinsic mechanism of apoptosis [6]. Both the intrinsic and extrinsic apoptotic mechanisms lead to the activation of caspase 3, which mediates apoptosis through nuclear activity.

Calcium (Ca^2+^) is a second messenger that influences the proliferation–apoptosis balance. Intracellular Ca^2+^ ([Ca^2+^]_i_) is modulated by receptor-operated, store-operated (SOC), and voltage-sensitive ion channels, ion exchangers, pumps, Ca^2+^ binding proteins, mitochondrial Ca^2+^ ([Ca^2+^]_m_), and endoplasmic reticulum (ER) and sarcoplasmic reticulum (SR) Ca^2+^ ([Ca^2+^]_ER_ and [Ca^2+^]_SR_) [7,8]. Intracellular Ca^2+^ release channels comprise one subset of ion channels; these include the ryanodine receptor (RyR) and inositol 1,4,5-triphosphosphate (IP_3_) receptor (IP_3_R) channels, both of which are localized to the ER and SR. RyR channels, which are activated by elevated [Ca^2+^]_i_ or protein signaling, and IP_3_R channels, which are activated by IP_3_ binding, release Ca^2+^ from the ER and SR. Through [Ca^2+^]_i_ signaling, these two channel types modulate muscle contraction and nerve impulse transmission [9,10]. Abberant Ca^2+^ transport from the ER or SR to the cytosol may elevate [Ca^2+^]_m_ and consequently induce mitochondrial dysfunction [11,12].

Beyond locomotion and neurotransmission, shifts in [Ca^2+^]_i_ homeostasis may also mediate cell death or proliferation. For instance, while [Ca^2+^]_i_ signaling via IP_3_R contributes to proliferation and oncogenesis, RyR [Ca^2+^]_i_ signaling supports apoptosis in lung cancer cells [10,13]. Furthermore, Ca^2+^ influx through T-type voltage-gated Ca^2+^ channels (VGCC) is implicated in the proliferation of cancerous and noncancerous cells, while the blockage of such channels promotes apoptosis in glioblastoma cells [14,15]. In contrast, Ca^2+^ influx through L-type VGCC causes death in bovine chromaffin cells [16]. Notably, [Ca^2+^]_i_-mediated cell death may be apoptotic or necrotic in nature, depending on the time of exposure and [Ca^2+^] _i_ involved [17].

Significantly, [Ca^2+^]_I_ signaling regulates proliferation, invasion, and metastasis in cancerous tissues [18]. A variety of oncologic therapies, including cisplatin, arsenic trioxide, trimethyltin chloride, and some candidate epigenetic drugs, induce their proapoptotic and anti-proliferative effects (in part or in whole) through the modulation of [Ca^2+^]_i_ [19,20]. Therefore, specific [Ca^2+^]_i_-affecting proteins, including transmembrane ion channels, which mediate Ca^2+^ flow between the extracellular space and the cytosol, are potential targets for chemotherapeutic agents.

Transient receptor potential (TRP) channels comprise a large family of membrane Ca^2+^ channels, which respond to a wide variety of environmental stimuli [21,22,23]. Transient receptor potential vanilloid 1, or vanilloid receptor 1 (TRPV1/VR1), known as the capsaicin receptor, is a member of the TRPV subfamily of TRP channels. TRPV1 is a ligand-gated ion channel which is activated by capsaicin and capsaicin analogues (e.g., resiniferatoxin, RTX), heat, and endogenous cannabinoids such as anandamide (AEA); its antagonists include capsazepine and ruthenium red [24,25]. The stimulation of TRPV1 causes Ca^2+^ and Na^+^ influx through transmembrane ion channels. While these channels generally exhibit selectivity for Ca^2+^ over Na^+^, the precise nature of this selectivity depends on a variety of factors, including the nature and concentration of the agonist [26]. TRPV1 is involved in thermoregulation, circadian rhythms, energy intake and metabolism, and acute, chronic, and inflammatory nociception; as such, the ion channel receptor is a target in the development of analgesic therapies [27,28,29,30,31,32]. Furthermore, given its role in modulating [Ca^2+^]_i_, TRPV1 influences the balance between proliferation and apoptosis [33]. This review aims to characterize the molecular mechanisms through which TRPV1 exerts the mentioned effect in the interest of identifying potential targets for anti-cancer drug development.

## 2. Expression of TRPV1 in Cancerous and Healthy Tissues

TRPV1 mRNA and protein are expressed in optic, pulmonary, nervous, cardiac, skeletal, circulatory, and skin cells, as well as in numerous cancer cell lines (Table 1). Compared to healthy cells, TRPV1 mRNA and/or protein expression levels are downregulated in many cancerous tissues, including colorectal, nervous system, endometrial, renal and skin cancers. However, TRPV1 mRNA levels are upregulated in the U373 glioblastoma line, high-grade astrocytes, “brain tumors,” and the RT4 renal cell carcinoma line; likewise, upregulated TRPV1 protein expression is observed in the U373 and RT4 cell lines (Table 2).

## 3. Balance Between Apoptosis and Proliferation Mediated by TRPV1

**Figure 1:**
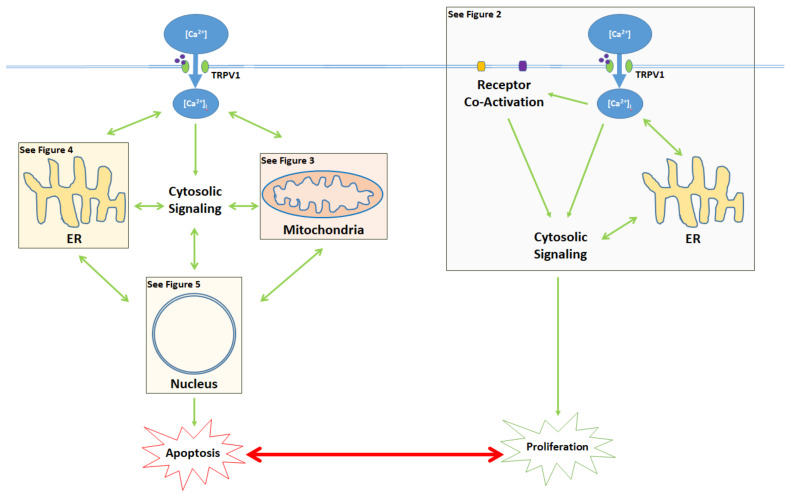
Activation of the TRPV1 ligand-gated ion channel causes Ca^2+^ influx into the cytosol and influences the balance between proliferation and apoptosis. Apoptotic signaling occurs through the cytosol, mitochondria, endoplasmic reticulum (ER), and the nucleus. In contrast, the proliferative effects of TRPV1 are mediated by the activation of other cell membrane receptors, ER signaling, and cytosolic protein signaling cascades. The proliferative, proapoptotic mitochondrial, proapoptotic ER, and proapoptotic nuclear signaling mechanisms are highlighted in the colored boxes, and specified in Figure 2 Figure 3 Figure 4 Figure 5, respectively.

The activation of the TRPV1 ion channel is a critical signal involved in numerous intracellular processes, some of which trigger either apoptosis or proliferation (Figure 1). While the apoptotic effects of TRPV1 are well characterized, literature on TRPV1-related proliferation remains sparse. The binding of exogenous agonists to the TRPV1 receptor and subsequent Ca^2+^ influx from the cytosol into the cell are characteristics shared between the apoptotic and proliferative pathways. However, both the positive allosteric modulation of cell membrane TRPV1 receptors and the activation of endoplasmic reticulum-localized TRPV1 channels are associated exclusively with the pro-apoptotic pathway [43,55].

## 4. TRPV1-Mediated Proliferation

**Figure 2:**
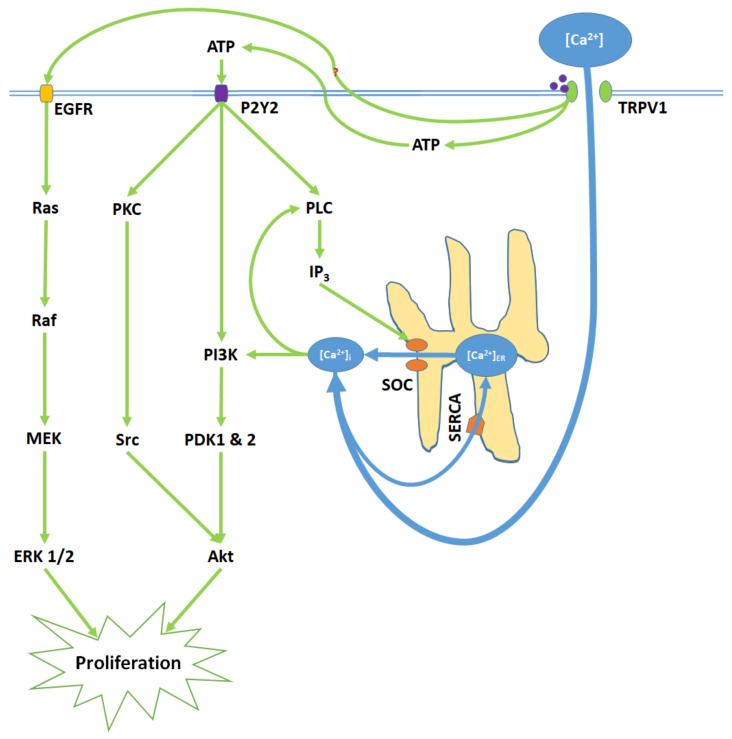
TRPV1 induces proliferation through Ca^2+^ entry, ATP release and membrane P2Y2 receptor activation, and the transactivation of epidermal growth factor receptor (EGFR). Elevated [Ca^2+^]_i_ and ATP-P2Y2 binding upregulate intracellular IP_3_ via phospholipase C (PLC); IP_3_ opens store-operated channels (SOC) and thereby causes Ca^2+^ release from the ER. Activated P2Y2 receptors also begin the PI3K/Akt pathway, a kinase signaling cascade that ultimately activates Akt. TRPV1 additionally transactivates EGFR; this prompts Ras/Raf/MAPK-ERK kinase (MEK)/extracellular signal-regulated kinase (ERK) signaling, which upregulates ERK 1/2 mitogen-activated protein kinases (MAPK). Akt and ERK 1/2 MAPK promote proliferation through nuclear activity.

TRPV1 promotes proliferation upon activation by capsaicin, glycolic acid, anandamide (AEA), and its analogue SKM 4-45-1 (Figure 2) [40,41]. Glycolic acid stimulates Ca^2+^ influx into the cell and corresponding elevation of [Ca^2+^]_i_ through TRPV1 channel opening. Furthermore, glycolic acid-TRPV1 interactions stimulate the release of intracellular ATP molecules into the cytosol, where they bind to the membrane G protein-coupled P2Y2 receptors [40]. Elevated [Ca^2+^]_i_ and stimulated P2Y2 receptors activate phospholipase C (PLC), resulting in the upregulation of intracellular IP_3_ levels and subsequent store-operated Ca^2+^ entry [56,57,58]. Elevated [Ca^2+^]_i_ and protein signals from the P2Y2 receptor activate the phosphoinisitide-3-kinase, PI3K [59,60]. PI3K subsequently activates the phosphoinositide-dependent kinases PDK 1 and 2. P2Y2 protein signals also activate protein kinase C (PKC), which activates the proto-oncogene protein kinase Src [61]. PDK 1 and 2 and Src then phosphorylate the Akt (serine/threonine protein kinase), promoting proliferation [59,60,61]. Concurrently, TRPV1 phosphorylates and thereby transactivates the epidermal growth factor receptor (EGFR) [62,63]. EGFR activates the Ras protein, a small GTPase, which in turn activates the serine-threonine protein kinase Raf; this kinase phosphorylates the mitogen-activated protein kinase (MAPK) kinases MEK 1/2. Finally, MEK 1/2 activate the extracellular signal-regulated kinases ERK 1/2 and their associated MAPKs, which further enhance proliferation [64].

Interestingly, in endothelial colony-forming cells (ECFC), capsaicin downregulates proliferation induced by AEA, potentially suggesting that the two TRPV1 agonists compete for binding sites and differentially influence the apoptosis–proliferation balance [41]. Additionally, despite its status as a TRPV1 antagonist, capsazepine upregulates proliferation in canine breast cancer cells [42] Another TRPV1 antagonist, AMG9810, enhances proliferation in human keratinocytes and murine skin cancer models [63]. As such, the precise functions of TRPV1 agonists and antagonists in proliferation may differ significantly between cell lines and therefore require further characterization (Table S1): ref. [35,41,42,62,63,65,66].

## 5. The Apoptotic Pathway and Upstream Cytosolic Effects

Apoptotic effects mediated by TRPV1 begin with the binding of the receptor to exogenous agonists or positive allosteric modulators, as well as the activation of the receptor through non-ligand means (such as magnetic fields). As TRPV1 is a ligand-gated cation channel, agonist binding results in Ca^2+^ and Na^+^ influx into the cell; notably, TRPV1 displays greater Ca^2+^ than Na^+^ affinity [67]. The mentioned Ca^2+^ influx elevates [Ca^2+^]_i_ (Table S2): ref. [34,35,37,38,39,43,46,48,49,50,54,55,65,68,69,70,71,72,73,74,75,76,77,78,79]. The co-application of TRPV1 antagonists, including capsazepine, ruthenium red (RR) and idioresiniferatoxin (I-RTX), attenuates agonist-induced Ca^2+^ influx; furthermore, capsazepine alone reduces [Ca^2+^]_i_ (Table S9): ref. [34,35,38,39,40,43,46,49,65,69,70,72,73,75,76,77,79].

## 6. Mitochondrial Pathway

The activation of TRPV1 by capsaicin leads to the phosphorylation and activation of the ATM serine-threonine kinase, which induces the downstream Fas pathway (Table S3): ref. [45,52]. ATM activation upregulates the Fas/CD95 death receptor, which co-clusters with TRPV1 to form a death signal complex. This function of this complex in TRPV1-mediated apoptosis is the cleavage of procaspase 8 into active caspase 8; caspase 8 transforms the BH3 interacting domain death agonist (BID) into its truncated form, which then contributes to the mitochondrial dysfunction [52]. The co-application of the TRPV1 antagonist capsazepine with capsaicin reduces the extent of TRPV1-Fas/CD95 co-clustering (Table S10): ref. [52].

**Figure 3:**
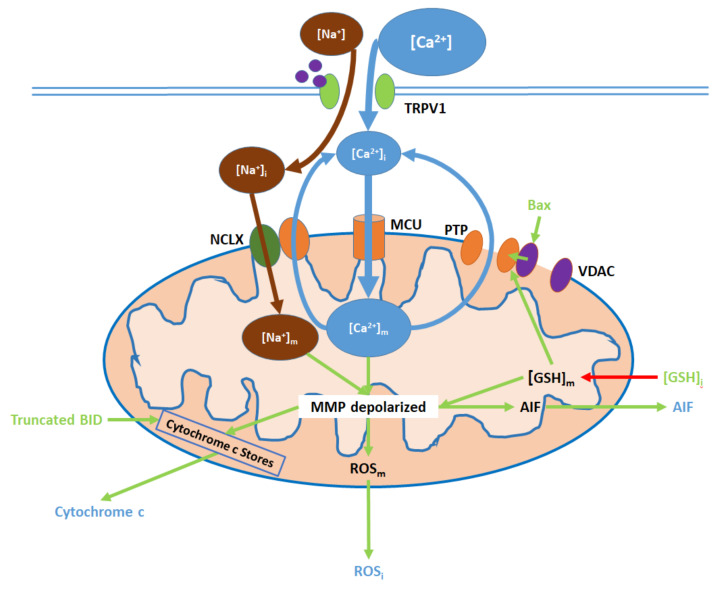
TRPV1 induces mitochondrial dysfunction through Ca^2+^ and Na^+^ entry, membrane depolarization, ROS production, and the release of cytochrome c and apoptosis-inducing factor (AIF). Initial Ca^2+^ and Na^+^ influx causes the hyperpolarization of the mitochondrial membrane, while consequent Ca^2+^ export through the permeability transition pore (PTP) and active Ca^2+^ removal via the NCLX depolarize the membrane. As inputs, downregulated [GSH]_i_ and upregulated Bax arise from nuclear activity. Upon their release (driven by membrane depolarization), AIF translocates directly to the nucleus, cytochrome c participates in intracellular caspase 9 activation, and intracellular ROS (ROS_i)_ supports the activation of p38 MAPKs.

Elevated [Ca^2+^]_i_, resulting from TRPV1 channel opening, causes an initial increase in [Ca^2+^]_m_ and subsequent downstream effects, which can be attenuated by TRPV1 antagonists (Figure 3; Table S10): ref. [38,39,45,49,55,65,69,72,77]. Ca^2+^ entry into the mitochondrial matrix is mediated by the mitochondrial Ca^2+^ uniporter (MCU) [74,80]. To maintain mitochondrial homeostasis, some of the Ca^2+^ returns to the cytosol via the mitochondrial membrane Na^+^/Ca^2+^ exchanger (NCLX), which also transports Na^+^ from the cytosol into the matrix [74]. Additionally, reductions in intracellular glutathione ([GSH]_i_) levels (mediated by ER and nuclear action) decrease mitochondrial glutathione ([GSH]_m_) levels and mitochondrial ROS (ROS_m_) production, while Bax is upregulated via nuclear action; both GSH depletion and Bax binding to mitochondrial membrane voltage-dependent anion channels (VDAC) promote the opening of the mitochondrial permeability transition pore (PTP), through which further Ca^2+^ exit to the cytosol occurs [81,82]. Finally, Na^+^, which entered the matrix via the NCLX, is exported by the sodium–hydrogen exchanger (NHE), which also imports protons [83,84].

Initial elevations in [Ca^2+^]_m_ and [Na^+^]_m_ cause hyperpolarization of the membrane potential, followed by the depolarization upon PTP opening [85]. [GSH]_m_ reduction contributes to mitochondrial membrane depolarization, which in turn upregulates ROS_m_ generation. Intracellular ROS (ROS_i_) levels increase due to ROS_m_ export to the cytosol [82,86]. Membrane depolarization also prompts the release of apoptosis-inducing factor (AIF) into the cytosol [87]. Furthermore, in conjunction with truncated BID activity, membrane depolarization promotes the export of cytochrome c from mitochondrial stores into the cytosol [52,87].

In the cytosol, ROS_i_ upregulates p38 and associated MAPKs, which upregulate caspase 9 and contribute to ER stress [53]. ROS_i_ levels are also elevated in the presence of Nerve Growth Factor [53]. Cytosolic cytochrome c upregulates caspase 9 activity while AIF translocates to the nucleus, binds to DNA, and induces DNA fragmentation and condensation [80,88]. See Table S4. ref. [38,39,45,47,49,52,55,65,72,74,77,87,89] for details.

## 7. Endoplasmic Reticulum (ER) Pathway

**Figure 4:**
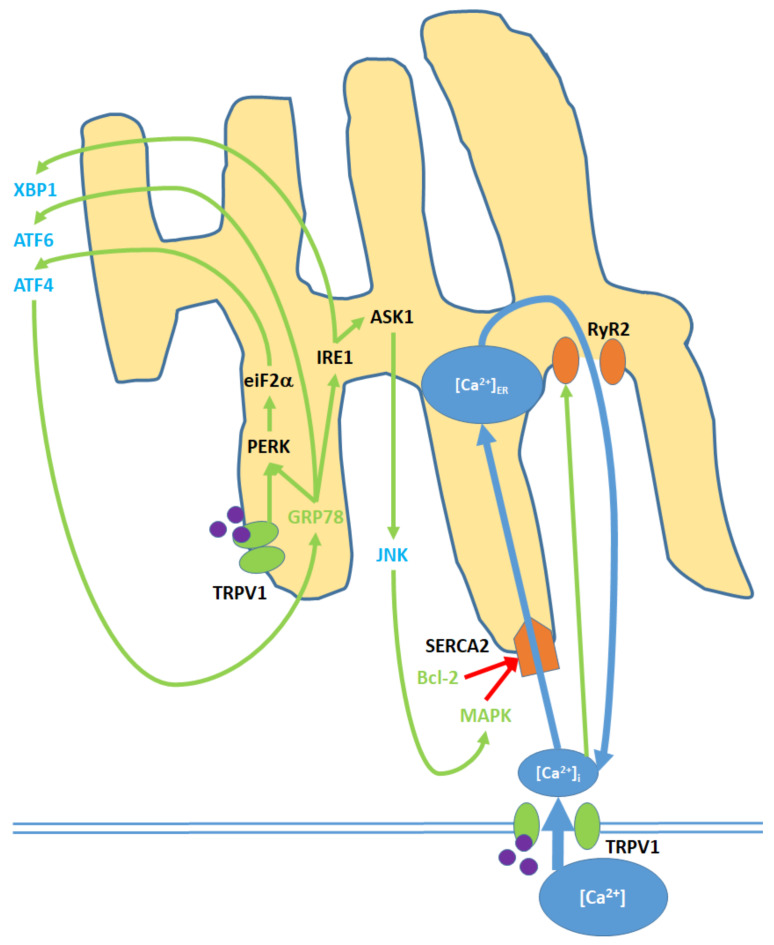
Cell membrane and ER TRPV1 activation promote ER stress through the modulation of [Ca^2+^]_ER_, activation of various kinases, the upregulation of nuclear transcription factors, and the release of JNK into the cytosol. TRPV1 proteins localized to the ER membrane contribute only to protein signaling within the ER, while TRPV1 channels in the cell membrane promote both [Ca^2+^]_i_ and protein signaling. Initial Ca^2+^ entry into the ER occurs through the SERCA2 pump, which is eventually blocked, causing net Ca^2+^ export via the RyR2 channels. GRP78 upregulation and Bcl-2 downregulation, as inputs, arise from nuclear activity. MAPK is both an input and output of ER stress, as it is upregulated via both mitochondrial activity and c Jun N-terminal kinases (JNK). ATF4, ATF6, and XBP1 are transcription factors that constitute the downstream nuclear targets of ER stress; ATF4, in particular, feeds back to the ER by upregulating GRP78.

Within the pro-apoptotic pathway, the ER is an intracellular signaling center that modulates nuclear transcription factors, [Ca^2+^]_i_ and kinase activity (Figure 4; Table S5): ref. [43,45,49,73,78,87,89]; Table S11: ref. [49,78]. Elevated [Ca^2+^]_i_ promotes the activity of the SERCA(2) pump, which facilitates Ca^2+^ entry into the ER/SR [90]. To maintain homeostasis given elevated [Ca^2+^]_ER_, Ca^2+^ is exported to the cytosol via the RyR2 (ryanodine receptor (2) channels, which are activated by increases in [Ca^2+^]_i_ [91]. As discussed for the mitochondria, increases in ROS_i_ promote the upregulation of (p38) MAPK. MAPK, along with decreased Bcl-2 levels resulting from nuclear activity, causes the blockage of the SERCA(2) pump over time and therefore decreases cytosol-ER Ca^2+^ transfer [92].

Upon activation by endogenous agonists, TRPV1 protein units localized to the ER activate the eukaryotic translation initiation factor, eiF2-α, which promotes expression of activating transcription factor 4 (ATF4) [43,78]. ATF4, through nuclear activity, induces the expression of GRP78, or binding immunoglobin protein, which translocates to the ER and activates the endoplasmic reticulum protein kinase PERK; in turn, PERK further upregulates eiF2- α [93]. Activating transcription factor 6 (ATF6) and inositol requiring enzyme 1 (IRE1) are also upregulated by GRP78 [87]. IRE1, in turn, upregulates the apoptosis signal-regulating kinase, ASK1, which upregulates the c Jun N-terminal kinases (JNK) and prompts their release into the cytosol [94]. Cytosolic JNK contributes to MAPK upregulation [45]. IRE1 also upregulates X-box binding protein 1 (XBP1), a transcription factor [89].

## 8. Nuclear and Downstream Cytosolic Effects

**Figure 5:**
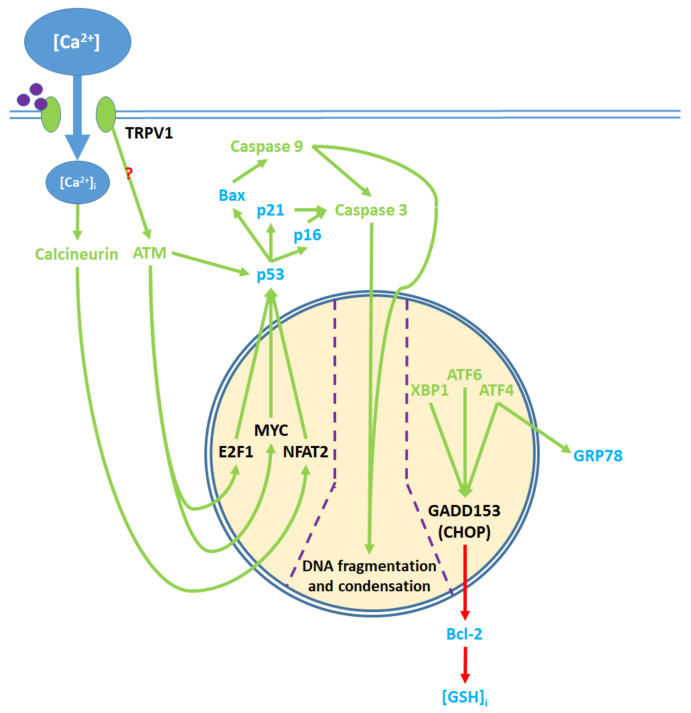
Pro-apoptotic processes induced by TRPV1 localized in the nucleus. The nuclear component of ER stress occurs as ATF4, ATF6, and XBP1 are activated by ER stress and upregulate GADD153, which in turn downregulates Bcl-2 protein production. The upregulation of GRP78 by ATF4 feeds back to and enhances ER stress. The cytosolic activation of the ATM serine-threonine kinase by TRPV1 protein signaling and calcineurin by elevated [Ca^2+^]_i_ promote the nuclear transcription factors E2F1, MYC, and NFAT2, which upregulate p53. The precise mechanism through which TRPV1 activates ATM remains unclear. p53 upregulates the apoptotic mediators Bax, p16, and p21, which activate caspase 9 and 3. Activated caspases translocate from the cytosol to the nucleus, where they mediate DNA fragmentation and condensation.

Upstream proapoptotic nuclear activity results from transcription factor activation via cytosolic and ER protein signaling (Figure 5). [Ca^2+^]_i_ elevation promotes the activation of calcineurin, a protein phosphatase [48,54]. Calcineurin upregulates the NFAT2 transcription factor while cytosolic ATM activation upregulates the myc proto-oncogene (MYC) and E2F1 transcription factors [45,52]. Together, NFAT2, MYC, and E2F1 upregulate the tumor suppressor protein p53 [48,52]. ATM also phosphorylates p53 in a nuclear activity-independent manner [95,96]. Activated p53 upregulates the cyclin dependent kinase (Cdk) inhibitors p16 and p21, and the proapoptotic Bcl-2 family protein Bax [48,54,71,87].

Through ER action, the transcription factors XBP1, ATF4 and ATF6 are upregulated. ATF4 complexes with another transcription factor, ATF1, to upregulate GRP78 protein expression [93]. XBP1, ATF4, and ATF6 together upregulate the GADD153/CHOP transcription factor, which in turn downregulates Bcl-2 protein expression [78,89]. Decreased Bcl-2 levels lead to [GSH]_i_ depletion [82]. In notable contrast to this model, the stimulation of TRPV1 receptors with capsaicin upregulates Bcl-2 in RT4 bladder cancer cells [52].

Bax protein, which is upregulated by nuclear activity, and [GSH]_i_, which decreases, regulate PTP opening in the mitochondria [81,82]. The downregulation of Bcl-2 and upregulation of GRP78 contribute to ER stress [92,93]. Together, Bax, p16, and p21 promote caspase activity [87,97,98]. Caspase 9 activates caspase 3. Caspase 3 further contributes to the downregulation of Bcl-2 by converting Bcl-2 into the Bax-like analogue Bcl-2ΔN34 [99]. With some exceptions as discussed above, TRPV1 agonists upregulate these proapoptotic nuclear signaling mechanisms, while antagonists of the receptor downregulate said mechanisms (Table S6): ref. [45,48,52,54,71,78,87,89,100]; Table S12: ref. [78].

Caspase activity is well characterized in relation to TRPV1-mediated apoptosis (Table S7): ref. [45,47,48,49,52,54,55,65,69,71,72,73,75,77,78,100,101]; Table S13: ref. [49,55,65,72,75,77]. Activated caspase 9 and 3 interact with DNA in the nucleus and cause DNA fragmentation and condensation [39,45,49,100]. Subsequently, apoptosis ensues (Table S8): ref. [34,35,36,38,39,43,45,47,48,49,50,54,55,65,71,72,75,76,77,78,79,100,101,102]; Table S14: ref. [34,38,39,43,45,49,54,55,65,71,75,77,78,79].

## 9. TRPV1 as a Potential Target for Anti-Cancer Therapies

The collective objective of oncologic therapies is to restore and maintain a homeostatic balance between proliferation and apoptosis. In this interest, many anti-cancer compounds induce and enforce apoptosis through the targeting of p53 [103]. The accumulation of p53 amplifies the tumor suppressor protein’s downstream apoptotic and anticarcinogenic effects in vitro (e.g., HCT116 cell line) and in vivo (e.g., mice) [104]. Furthermore, independently of p53, some anti-cancer drugs bind directly to and damage DNA, inhibiting transcription and therefore downregulating proliferation [105]. While the modulation of individual components of the intrinsic apoptotic and proliferative mechanisms can constitute an effective means of tumor suppression, enormous potential exists for therapies that regulate both mechanisms to shift the balance.

In this regard, [Ca^2+^]_i_, a universal second messenger regulating cell death and survival, is a promising target. [Ca^2+^]_i_ signaling is highly versatile, as it influences intracellular pathways for proliferation, differentiation, and apoptosis in neuroblastoma cells [106]. Within cancerous tissues, the molecular machinery involved in [Ca^2+^]_i_ signaling is modified to promote proliferation and minimize apoptosis. Modifications of this nature may downregulate Ca^2+^ entry into the cytosol (e.g., blockade of Ca^2+^ release from ER stores) or eliminate downstream targets of [Ca^2+^]_i_ signaling (e.g., enforced loss of p53) [107].

Numerous existing anti-cancer therapies, including metal compounds, anti-metabolites, and various natural and synthetic organic molecules, disrupt [Ca^2+^]_i_ homeostasis (via elevation of [Ca^2+^]_i_ due to transmembrane Ca^2+^ influx and release of Ca^2+^ from intracellular stores) and thereby promote apoptosis [8,108,109,110]. As such, [Ca^2+^]_i_ affects mitochondrial dysfunction and ER stress along the proapoptotic pathway. [Ca^2+^]_i_ is also an important regulator of the PI3K/Akt pathway, which promotes the proliferation of cancer cells [60].

TRPV1, a ligand-gated ion channel, modulates [Ca^2+^]_i_, [Ca^2+^]_m,_ and [Ca^2+^]_ER_. While TRPV1 is well characterized with regards to cell death, the link between TRPV1 and proliferation has yet to be thoroughly investigated. The vast majority of studies pertaining to cell death elucidated a connection between TRPV1 activation and apoptosis in both cancerous and benign cell lines. However, some found TRPV1 activation in breast cancer cells (MCF-7) to result in necrotic cell death [102].

Notably, TRPV1 is expressed at the mRNA and protein levels in a wide variety of cancerous and non-cancerous cell lines (Table 1). In numerous nervous, colorectal, endometrial, renal, and dermal cancer cell lines, TRPV1 expression is reduced in comparison with healthy cells (Table 2). This pattern may indicate a primarily pro-apoptotic role for TRPV1 in the tumors. Under these conditions, the upregulation and subsequent stimulation of TRPV1 can potentiate the innate apoptotic pathway. It is therefore relevant that capsaicin upregulates the mRNA and protein expression of TRPV1 in certain healthy and cancerous (e.g., nasopharyngeal and skin cancer) cell lines [35,54,87]. In contrast, TRPV1 expression levels in high-grade astrocytes, “brain tumors,” U373 cells, and RT4 renal cell carcinoma cells are elevated in comparison to healthy controls (Table 2). These findings may hint that TRPV1 contributes primarily to proliferation rather than apoptosis in the cancerous cell lines in which its expression is upregulated. To combat carcinogenesis in these cases, it would be necessary to shift the TRPV1-mediated balance towards apoptosis, while native TRPV1 expression levels may be sufficient to induce the desired apoptotic effects once the aforementioned shift is achieved.

Similarly, in the vast majority of cell lines, apoptosis is increased by TRPV1 agonists and downregulated by TRPV1 antagonists. However, in canine breast cancer cells, agonists and antagonists both support proliferation; in human breast cancer cells, both types of molecules support apoptosis [42,76]. These results suggest that the nature of the proliferation–apoptosis balance may differ between cell lines.

**Figure 6:**
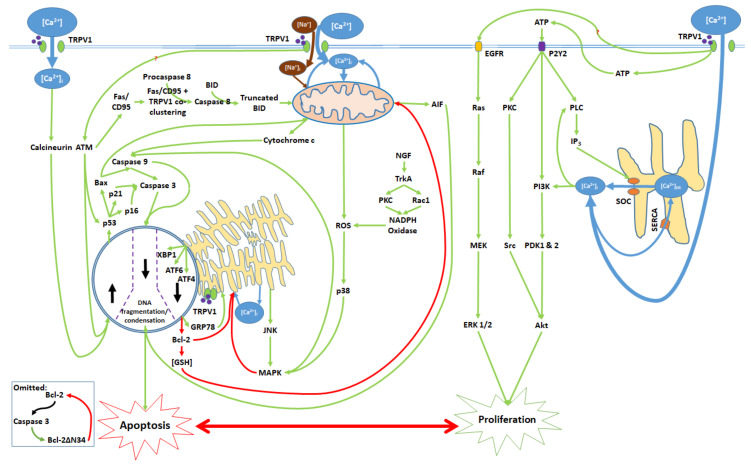
Activation of the TRPV1 ligand-gated ion channel and subsequent [Ca^2+^]_i_ and protein signaling influence the balance between proliferation and apoptosis. Mitochondrial dysfunction, ER stress, transcription factor activation, and nuclear activity promote caspase activation and the consequent DNA degradation, which are characteristic of apoptotic cell death. TRPV1 protein signaling upon activation induces the release of ATP, which binds to the P2Y2 receptor and activates a kinase signaling cascade, leading to ER Ca^2+^ release and Akt activation. TRPV1 also activates EGFR and its associated protein signaling pathway, which upregulates ERK 1/2. Akt and ERK 1/2 contribute to cell proliferation. All cytosolic signaling pathways associated with the proliferative and apoptotic effects of TRPV1 activation are shown.

The pathways through which TRPV1 channel activation affects apoptosis or proliferation are distinct and competitive (Figure 6). Signaling mechanisms that mediate the pro-apoptotic effects of TRPV1 act through the mitochondria, ER, nucleus, and cytosol. Ca^2+^ and Na^+^ influx into the mitochondria results in the depolarization of the mitochondrial membrane and subsequent release of cytochrome c, AIF, and ROS into the cytosol. Meanwhile, ER stress results in JNK release into the cytosol and the upregulation of the nuclear transcription factors ATF4, ATF6, and XBP1, which decrease intracellular Bcl-2 and [GSH]_i_. JNK and p38 (increased due ROS_i_) upregulate MAPK. Furthermore, TRPV1 [Ca^2+^]_i_ and protein signaling activates calcineurin and ATM, which act through transcription factors to upregulate the tumor suppressor gene p53. p53 subsequently upregulates the proapoptotic proteins Bax, p16, and p21. Finally, MAPK, cytochrome c, p16, p21, and Bax activate caspases. Caspase 9 activates caspase 3; the two caspases and AIF cause DNA fragmentation and condensation in the nucleus, and ultimately apoptosis [39,45,49,87,97,98,99,100].

The proliferative effects of TRPV1 activation are linked to ATP release into the extracellular space and transactivation of EGFR. ATP binding to cell membrane P2Y2 receptors starts an intracellular kinase signaling cascade that is enhanced by IP_3_-mediated Ca^2+^ release from ER stores and ultimately activates the Akt kinase; concurrently, EGFR activation prompts a separate series of protein signals to upregulate ERK 1/2. Both Akt and ERK 1/2 enhance proliferation. Akt is a serine/threonine protein kinase with oncogenic effects. The localization of active Akt to the cell membrane results in oncogenic transformation of chicken embryonic fibroblasts and hemangiosarcoma development in young chickens [111]. The linking of TRPV1 and P2Y2 signals to cell proliferation and oncogenicity suggests TRPV1 as a potential target for anti-cancer therapies, in the interest of downregulating proliferative protein signaling. As was demonstrated by Huang et al., the activation of TRPV1 promotes the proliferation and migration of esophageal squamous cell carcinoma cells. While capsaicin activates TRPV1, a TRPV1 inhibitor (AMG9810) antagonizes its effects [66].

TRPV1 mediates apoptosis primarily through [Ca^2+^]_i_ and protein signaling; in contrast, the channel stimulates proliferation via ATP release, P2Y2 receptor activation, and EGFR transactivation, with a limited role for [Ca^2+^]_i_. As such, the activation of TRPV1 in the absence of P2Y2 and EGFR activation may result in the dominance of the apoptotic pathway. Exogenous and endogenous TRPV1 agonists, applied in conjunction with P2Y2 and EGFR antagonists, may therefore constitute a potential form of anti-cancer therapy.

Given the role of P2Y2 in activating PI3K and beginning the PI3K/Akt pathway, inactivation of the receptor may have implications in oncologic drug discovery. PI3K/Akt signaling is firstly implicated in cancer cell proliferation and tumorigenesis [112,113,114]. More importantly, the PI3K/Akt pathway is an important mediator of cancer drug resistance, such as that of breast cancer cells to trastuzumab [115]. As such, blockade of P2Y2 receptors has the potential to reduce PI3K activation and thereby impede the development of cancer drug resistance.

The inhibition of EGFR activity may likewise support effects to curb cancer drug resistance. EGFR is a transmembrane growth factor receptor which contributes to cell proliferation via the Ras/Raf/MEK/ERK signaling pathway [64]. This pathway promotes the resistance of hematopoietic cells to doxorubicin, and acts in conjunction with PI3K/Akt signaling in support of tumorigenesis [116]. Notably, functional crosstalk occurs between the PI3K/Akt and Ras/Raf/MEK/ERK pathways in colon cancer cells, as the downregulation of one pathway corresponds with the upregulation of the other; however, treatment with both patritumab (a PI3K inhibitor) and trametinib (an MEK inhibitor) reduces the viability of said cells [117]. Electroacupuncture (EA) therapy may be a viable avenue for the dual inhibition of PI3K/Akt and Ras/Raf/MEK/ERK signaling; recent evidence suggests that EA in the cerebellum downregulates PI3K, Akt, PKC, and ERK [31]. In this light, the activation of TRPV1 in the presence of PI3K/Akt and Ras/Raf/MEK/ERK pathway inhibitors—potentially including P2Y2 and EGFR antagonists—may promote cancer cell apoptosis through [Ca^2+^]_i_ signaling without the induction of proliferative mechanisms.

Interestingly, PKC, a protein kinase, is involved in both the proliferative and apoptotic pathways. The activation of PKC as part of the Nerve Growth Factor pathway supports the elevation of ROS_i_ levels via nicotinamide adenine dinucleotide phosphate (NADPH) oxidase upregulation [53,118]. However, PKC also activates the Src proto-oncogene, which promotes cell proliferation. Therefore, PKC and NGF have dual roles as determinants of cell fate. In the development of anti-cancer drugs, the presence or absence of NGF must be taken into account, as despite the inactivation/antagonism of the P2Y2 receptor, NGF may nevertheless stimulate cell proliferation [119].

It is important to note that TRPV1 modulates the apoptosis–proliferation balance through mechanisms beyond [Ca^2+^]_i_ signaling and may therefore exert pleiotropic effects in cancerous tissues. In particular, the implications of TRPV1 for inflammation are multi-faceted (Figure S1). The activation of the membrane ion channel by formaldehyde and particulate matter (in an asthmatic murine model), and acidic solution (in human esophageal cells) enhances inflammation. Along this pro-inflammatory axis, TRPV1 induces the release of substance P and calcitonin gene-related peptide (CGRP), both of which are pro-inflammatory neuropeptides [120]. Substance P and CGRP activate the neurokinin 1 (NK1R) and CGRP (CGRPR) receptors, respectively, and promote the release of pro-inflammatory cytokines such as tumor necrosis factor alpha (TNF-α) and interleukins 1-beta (IL-1β), 6 (IL-6), and 8 (IL-8) [121,122]. These cytokines induce vasodilation and ultimately inflammation; significantly, inflammation is associated with cell proliferation and tumorigenesis [123,124].

In contrast, TRPV1 activation by capsaicin attenuates pro-inflammatory cytokine release and inflammation [120,125]. Within this anti-inflammatory mechanism, TRPV1 stimulation prompts the release of somatostatin (SST), an anti-inflammatory neuropeptide which binds to SST receptor 4 (sst_4_) and downregulates the aforementioned pro-inflammatory cytokines [126]. SST also attenuates substance P and CGRP release; this further downregulates said cytokines [127]. Capsaicin, through TRPV1, thereby decreases inflammation and may reduce inflammation-related cell proliferation.

TRPV1 is expressed in a wide variety of immune cells, such as macrophages, neutrophils, T cells, and dendritic cells [128]. As such, in attempting to upregulate apoptosis through TRPV1, care must be taken to prevent the inadvertent promotion of proliferation through inflammation. Further research is necessary to fully elucidate the dependence of TRPV1’s inflammatory effects on agonist type, cell/cancer type, and tumor microenvironment. Based on current knowledge, capsaicin and nonivamide are anti-inflammatory agents that exert their effects through TRPV1 channel activation [129,130]. These TRPV1 agonists may therefore have dual effects in upregulating apoptosis through [Ca^2+^]_i_ signaling while suppressing proliferation through inflammation reduction.

In the development of anti-cancer drugs to affect TRPV1, it is in all cases necessary to first characterize the apoptosis–proliferation balance that exists in the target cell line, taking into account factors such as inflammation. Depending on the balance, oncologic therapies may upregulate and stimulate TRPV1 with carefully selected agonists, downregulate or otherwise inhibit TRPV1, or target specific aspects of the TRPV1-mediated mechanisms to enhance the protein’s apoptotic effects. Recent molecular and clinical developments suggest that these objectives are achievable.

Cancer management based on the targeting of TRPV1 currently encompasses a wide range of compounds with synthetic as well as natural origins. Arvanil, a synthetic TRPV1 agonist, decreases tumor weight and improves survival time in mice with implanted gliomas [43]. Moreover, bisphenol A (BPA) is a synthetic compound whose presence in the environment and in human tissues results from its widespread use and biological accumulation. BPA has hormone-like properties and can mimic estrogen and interact with its receptors; it can thereby modulate cell proliferation, apoptosis, and migration and contribute to carcinogenesis [131]. However, the proliferative and oxidative effects of BPA, enhanced through TRPV1 activation, are reduced by sodium selenite (Na-Se), which attenuates BPA-induced increases in cell number, mitochondrial oxidative stress, and modulation of TRPV1 channel activity in MCF-7 cells [132]. Interestingly, despite the function of both capsaicin and 6-gingerol as TRPV1 agonists, the two compounds exhibit opposing effects on cancerous cells. In a urethane-induced lung carcinogenic model, capsaicin enhances proliferation and epithelial-mesenchymal transitions via a decrease in TRPV1 expression and an increase in EGFR, followed by decreases in nuclear factor-κB (NF-κB) and cyclin D1. On the other hand, 6-gingerol reverses the carcinogenic effects of capsaicin by increasing TRPV1 expression and decreasing EGFR, NF-κB and cyclin D1 [133].

TRPV1 channels also modulate the efficacy of existing anti-cancer therapies. Alpha-lipoic acid and selenium enhance the cytotoxic efficacy of cisplatin, an existing platinum-derived anti-cancer drug, via TRPV1 stimulation [134,135]. Moreover, TRPV1 is differentially expressed in the bladder cancer cell lines 5637 and T24. TRPV1 mRNA and protein expression is low in T24 and high in 5637 cells; the activation of TRPV1 by capsaicin inhibits the growth of 5637 cells. Moreover, the activation of TRPV1 promotes the anti-proliferative efficacy of pirarubicin, an anthracycline agent used for intravesical chemotherapy of superficial bladder cancer. Therefore, TRPV1 stimulation may represent a strategy to increase the efficacy of traditional chemotherapeutic agents against bladder cancer [136]. In addition, the anticancer activity of selenium via TRPV1 was evaluated in the MCF-7 breast cancer cell line. Increases in mitochondrial membrane depolarization, apoptosis, and caspase 3 and caspase 9 levels occur after treatment with selenium alone and in combination with cisplatin. Therefore, the interaction of selenium and cisplatin with the same intracellular cascade through modulation of TRPV1 can bring about remarkable advantages in oncology [135]. Similarly, melatonin supports the anti-cancer effects of the chemotherapeutic agent doxorubicin via TRPV1 activation and subsequent apoptosis in MCF-7 cells [137]. It is notable that doxorubicin treatment may cause cardiomyopathy; the inhibition of the proapoptotic Bax protein is a potential avenue through which to prevent this side effect [138]. On the other hand, the antioxidant plant *Hypericum perforatum* (HP) does not support the antitumor effects of 5-Fluorouracil through TRPV1 modulation. The apoptotic effects of 5-Fluorouracil, mediated by TRPV1 activation in MCF-7 cells, are downregulated by treatment with HP [139].

Moreover, fibulin-5 is a multifunctional extracellular matrix protein with lower expression in colorectal cancer tissues than in peritumoral areas. Given its role in promoting apoptosis through TRPV1 downregulation and consequent ROS/MAPK and Akt signaling, fibulin-5 is a potential novel target in the treatment of colorectal cancer [140]. Furthermore, the endocannabinoid/endovanilloid system, composed of two G-protein coupled cannabinoid receptors (CB1 and CB2) and TRPV1, and their respective ligands and enzymes, is targeted in a novel therapeutic approach for T-cell acute lymphoblastic leukemia (T-ALL). Treatment of T-ALL lymphoblasts with the selective CB2 agonist JWH-133 and TRPV1 agonist RTX produces pro-apoptotic and anti-proliferative effects [141].

### Recent Trends and Innovations in Oncologic Approaches Targeting TRPV1

Nanomedicine is associated with progress in anti-cancer research [142]. Distressing toxic effects are common consequences of chemotherapeutics. To mitigate this and other obstacles (e.g., poor biocompatibility, premature drug leakage, off-targeting), a [Ca^2+^]_i_ signaling cascade for cancer therapy via photothermal TRPV1 activation has been developed. This cascade creates artificial Ca^2+^ overload stress, causing cell death via mitochondrial dysfunction (involving caspase 3 and cytochrome c upregulation, and Bcl-2 and ATP downregulation) in vitro. Given TRPV1 overexpression and near-infrared (NIR) irradiation at tumor sites, a CuS@CaCO_3_-PEG nanoplatform can initiate the [Ca^2+^]_i_ cascade in both cancerous and non-cancerous tissues. Additionally, photothermal CuS nanoparticles involved in the nanoplatform allow for three-dimensional photoacoustic imaging in vivo [143]. Furthermore, an apoptosis-inducing TRPV1 nanoagonist comprised of semiconducting polymer nanoparticles (SPNs) as photothermally responsive nanocarriers and capsaicin as the agonist, has been developed. Under NIR irradiation, the nanoagonist releases capsaicin to activate cell membrane TRPV1 channels. Ionic influx into the mitochondria is followed by apoptosis in TRPV1-positive cancer cells. This photothermal mechanism allows for the release of high concentrations of TRPV1 agonist(s) at specific tumor sites with low systemic dosages [144]. Finally, nanoscale drug delivery systems based on single-walled carbon nanotubes can utilize TRPV1 channels for transmembrane drug transport [145].

Looking forward, natural products and drug conjugates can be evaluated for the treatment of prostate cancers with TRPV1 overexpression. The stoichiometric combination of a TRPV1 agonist with a small, positively charged cytotoxic agent constitutes a promising avenue for prostate cancer treatment [146]. Another potential oncologic approach utilizes a gold nanorod-assisted NIR light-activated tool to open TRPV1 channels and thereby induce apoptosis [147].

Beyond its potential as a therapeutic target, TRPV1 also has prognostic significance. It is, for instance, a biomarker in invasive breast carcinoma [148]. Moreover, TRPV1, alone and in combination with the Phosphatase and Tension Homolog (PTEN), is an important prognostic factor in epithelial ovarian and cervical cancers [149,150]. Finally, a non-invasive cancer detection method could utilize magnetoencephalography to measure cellular ion transport. In TRPV1-expressing HEK-293 cells, capsaicin induces a sudden change in the magnetic field signal, consistent with Ca^2+^ influx [151].

Targeting of TRPV1 represents an important avenue for cancer management, and current progress in related oncologic strategies is promising. However, the exact mechanisms by which the proliferation–apoptosis balance shifts in the described cases remain unclear; further investigations are therefore necessary to produce clinically applicable results.

## 10. Conclusions and Outlook

TRPV1, a ligand-activated membrane ion channel, functions in both apoptotic cell death and proliferation. Mitochondrial dysfunction and membrane depolarization, ER stress, caspase activation, and DNA damage are all implicated in TRPV1-mediated apoptosis. In contrast, TRPV1 supports proliferation through the activation of P2Y2 and EGFR, and the resulting intracellular protein signaling cascades. In healthy cells, a delicate and dynamic balance exists between the proliferative and apoptotic mechanisms. This balance is shifted in cancerous cells and tissues, in which proliferation dominates; however, the precise factors that modulate the balance remain largely uncharacterized.

As the objective of anti-cancer therapies is to hinder tumor growth through the enforcement of apoptosis and the minimization of proliferation, the TRPV1 ion channel constitutes a promising target. TRPV1 is constitutively expressed in a wide variety of cancerous cell lines, and the receptor’s differential expression in cancerous and healthy tissues provides insights into its role in the proliferation–apoptosis balance. Oncologic agents that target TRPV1 will exert their proapoptotic effects primarily through the modulation of [Ca^2+^]_i_, which has a dual role in that it regulates both proliferative and apoptotic mechanisms. Notably, the influence of TRPV1 on the PI3K/Akt signaling pathway may hold implications for the circumvention or minimization of cancer drug resistance. The activation of TRPV1 by agonists, in conjunction with a blockade of P13K/Akt signaling, may have therapeutic potential through the elevation of [Ca^2+^]_i_ and consequent upregulation of the apoptotic pathways. The parallel involvement of TRPV1 in proliferation and apoptosis enhances the receptor’s relevance as an oncologic target. Several innovative anti-cancer strategies targeting TRPV1 are currently in development.

## Figures and Tables

**Table 1:** mRNA and protein expression of the transient receptor potential vanilloid 1 (TRPV1) ligand-gated ion channel in a variety of cell lines. TRPV1 receptor expression is well characterized in the nervous and optic systems and less so in the muscular and skeletal systems. Cancerous cells lines are highlighted in gray.

Localization/Cancer Type	Cell Line/Source	TRPV1 mRNA Expression	TRPV1 Protein Expression	Source
Non-Cancer				
Eyes	Whole retina, Sprague-Dewley rats	Yes	Yes	[34]
	Retinal RGC, Sprague-Dewley rat	Yes	Yes	[34]
	Primary retinal RGC, Sprague-Dewley rat	Yes	Yes	[34]
	Whole retina, DBA/2 mice	--	Yes	[34]
	Whole retina, C57 mice	--	Yes	[34]
Lung	ASMC, Sprague-Dewley rats	Yes	Yes	[35]
	ASMC, chronic asthmatic Sprague-Dewley rats	Yes	Yes	[35]
Nervous System	Cortical neuron, Wistar rat	Yes	Yes	[36]
	Brain, Sprague-Dewley rat	--	Yes	[34]
	Brain, C57 mouse	--	Yes	[34]
	Type 1 SGZ NPC, p7-21, murine	Yes	Yes	[37]
	Type B SVZ NPC, p7-p21, murine	Yes	Yes	[37]
Heart	H9C2	Yes	Yes	[38]
Joints	Synoviocytes, Wistar rat	Yes	--	[39]
Skin	Epidermis, human skin	--	Yes	[40]
	In-vitro Reconstructed Skin Equivalent Model	--	Yes	[40]
Circulatory/Endothelium	ECFC	--	Yes	[41]
	EA.hy926	--	Yes	[41]
Cancer				
Breast Cancer	MCF-7	--	Yes	[42]
	CF.41	--	Yes	[42]
Nervous System Cancer	GL261	--	Yes	[43]
Leukemia	Jurkat	--	Yes	[44]
Renal Cell Carcinoma	786-O	Yes	Yes	[45]
Bladder Cancer	T24	Yes	Yes	[45]
	5637	Yes	Yes	[45]
Prostate Cancer	LNCaP	Yes	Yes	[46]
	PC-3	Yes	Yes	[46]
Sarcoma	Meth A	Yes	Yes	[47]
	CMS5	Yes	Yes	[47]

**Table 2:** mRNA and protein expression levels of the TRPV1 ligand-gated ion channel in cancerous cell lines, as compared to healthy tissues. The “TRPV1 mRNA vs. Normal” and “TRPV1 Protein vs. Normal” columns evaluate the mRNA and protein expression levels, respectively, observed in the cancerous cell lines relative to the corresponding control/healthy cells. “NC” indicates “No Change” in expression between the normal and cancerous tissues. Cancerous cells lines are highlighted in gray.

Cancer Type	Cell Line/Source	TRPV1 mRNA vs. Normal	TRPV1 Protein vs. Normal	Normal Comparison	Source
Colorectal	Human CRC	--	Decreased	Human Colorectal Sample	[48]
Nervous System	U87	Decreased	Decreased	NHA	[49]
	U373	Increased	Increased	NHA	[49]
	FLS	Decreased	--	NHA	[49]
	FC1	Decreased	--	NHA	[49]
	High Grade Astrocyte	Increased	--	Low Grade Astrocyte	[43]
	“Brain Tumors”	Increased	--	“Tumor Free Brain”	[43]
Endometrial	Ishikawa	NC	Decreased	HFF-1	[50]
	Hec50co	NC	Decreased	HFF-1	[50]
Renal	Human RCC	Decreased	Decreased	Human Renal Sample	[51]
	RT4	Increased	Increased	NHUC	[52]
	TCCSUP	Decreased	Decreased	NHUC	[52]
	J82	Decreased	Decreased	NHUC	[52]
	EJ	Decreased	Decreased	NHUC	[52]
Pheochromocytoma	PC12	--	Decreased	Rat DRG	[53]
Melanoma	WM793B	NC	NC	NHEM	[54]
	WM35	Decreased	Decreased	NHEM	[54]
	1205Lu	Decreased	Decreased	NHEM	[54]
	451Lu	Decreased	Decreased	NHEM	[54]
	UACC 62	Decreased	Decreased	NHEM	[54]
	UACC 257	Decreased	Decreased	NHEM	[54]
	Hs 294T	Decreased	Decreased	NHEM	[54]
	A375	Decreased	Decreased	NHEM	[54]
	A2058	Decreased	Decreased	NHEM	[54]
	Sk-mel-5	Decreased	Decreased	NHEM	[54]
	Primary human melanoma	Decreased	Decreased	Human melanocytic nevus tissues	[54]
	Metastatic human melanoma	Decreased	Decreased	Human melanocytic nevus tissues	[54]

## Supplementary Materials

The following are available online at https://www.mdpi.com/1422-0067/21/11/4177/s1, Table S1: Proliferative effects of the exogenous TRPV1 agonists capsaicin and glycolic acid, the endogenous agonist AEA and its analogue, SKM-4-45-1, and the antagonists capsazepine and AMG9810; Table S2: [Ca^2+^]_i_ elevation induced by various endogenous and exogenous TRPV1 agonists and activators; Table S3: Effects of the TRPV1 agonist capsaicin on the Fas/CD95 cytosolic pathway; Table S4: Mitochondrial pathway effects induced by TRPV1 agonists; Table S5: ER effects induced by endogenous and exogenous TRPV1 agonists; Table S6: Nuclear effects of endogenous and exogenous TRPV1 agonists; Table S7: Effects of exogenous and endogenous TRPV1 agonists on caspase activity; Table S8: Downstream pro-apoptotic effects of endogenous and exogenous TRPV1 agonists and antagonists; Table S9: Effects of TRPV1 antagonists on transmembrane Ca^2+^ transport; Table S10: Effects of TRPV1 antagonists on mitochondrial dysfunction; Table S11: Effects of TRPV1 antagonists on ER stress; Table S12: Effects of TRPV1 antagonists on nuclear activity; Table S13: Effects of TRPV1 antagonists on caspase activity; Table S14: Effects of TRPV1 antagonists on agonist-induced apoptosis; Figure S1: Activation of TRPV1 modulates pro- and anti-inflammatory signaling pathways.

## Abbreviations

13-HODE	13-HydroxyOctaDecadEinoic acid
2-AG	2-ArachidonylGlycerol
AEA	Anandamide
Akt	Akt serine/threonine protein kinase
AIF	Apoptosis Inducing Factor
ASMC	Airway Smooth Muscle Cells
ATF1	Activating Transcription Factor 1
ATF3	Activating Transcription Factor 3
ATF4	Activating Transcription Factor 4
ATF6	Activating Transcription Factor 6
ATM	ATM serine-threonine kinase
ATP	Adenosine TriPhosphate
Bax	Bcl-2 associated X protein
Bcl-2	B-cell lymphoma 2
BID	BH3 Interacting-domain Death agonist
BPA	BisPhenol A
Ca^2+^	Calcium
[Ca^2+^]_i_	Intracellular Calcium Concentration
[Ca^2+^]_m_	Mitochondrial Calcium Concentration
[Ca^2+^]_ER_	Endoplasmic Reticulum Calcium Concentration
CaM	CalModulin
CB1	CannaBinoid receptor type 1
CB2	CannaBinoid receptor type 2
CBD	CannaBiDiol
Cdk	Cyclin dependent kinase
CGRP	Calcitonin Gene-Related Peptide
CGRPR	Calcitonin Gene-Related Peptide Receptor
CRC	ColoRectal Cancer
DMBA	7,12-DiMethylBenz[a]Anthracene
DNA	DeoxyriboNucleic Acid
DRG	Dorsal Root Ganglion
E2F1	E2F transcription factor 1
EA	ElectroAcupuncture
ECFC	Endothelial Colony Forming Cells
EET	EpoxyEicosaTrienoic acid
EGFR	Epidermal Growth Factor Receptor
eIF2	eukaryotic Initiation Factor 2
ER	Endoplasmic Reticulum
ERK	Extracellular signal-Regulated Kinase
FADD	Fas-Associated protein with Death Domain
Fas/CD95	Fas cell surface death receptor/Cluster of Differentiation 95
GADD153; DDIT3; CHOP	DNA-Damage Inducible Transcript 3; C/EBP HOmologous Protein
GSH	Glutathione
[GSH]_i_	Intracellular Glutathione Concentration
GRP78; BiP	Binding immunoglobulin Protein
HCEC	Human Corneal Epithelial Cells
HP	*Hypericum perforatum*
ICR	Institute for Cancer Research
IL-1β	InterLeukin 1β
IL-6	InterLeukin 6
IL-8	InterLeukin 8
IP_3_	Inositol triPhosphate
IRE1	Inositol-Requiring Enzyme 1
IRTX	IodoResiniferaToXin
JNK	c-Jun N-terminal Kinase
MAPK	Mitogen-Activated Protein Kinase
MCU	Mitochondrial Calcium Uniporter
Mdm2	Mouse double minute 2 homolog
MEK	MAPK-ERK Kinase
MET	(R)-METhanandamide
mRNA	messenger RiboNucleic Acid
MYC	Myc proto-oncogene
Na^+^	Sodium
[Na^+^]_i_	Intracellular Sodium Concentration
[Na^+^]_m_	Mitochondrial Sodium Concentration
NADA	*N*-Arachidonoyl DopAmine
NADPH	Nicotinamide Adenine Dinucleotide PHosphate
NFAT2	Nuclear Factor of Activated T-cells 2
NF-κB	Nuclear Factor-Kappa light chain enhancer of activated B cells
NGF	Nerve Growth Factor
NHA	Normal Human Astrocytes
NHBE	Normal Human Bronchial Epithelial cells
NHEM	Normal Human Epidermal Melanocytes
NHUC	Normal Human Urothelial Cells
NIR	Near InfraRed
NK1R	NeuroKinin 1 Receptor
NPC	Neural Progenitor Cells
(m)NPC-CM	(murine) Neural Progenitor Cell-Culture Media
p16; CDKN2A	Cyclin-Dependent Kinase Inhibitor 2A
p21; CDKN1(A)	Cyclin-Dependent Kinase Inhibitor 1(A)
P2Y2	P2Y purinoceptor 2
p38 (MAPK)	p38 (Mitogen Activated Protein Kinase)
p53	tumor protein p53
PAM	Positive Allosteric Modulator
PCOS	PolyCystic Ovary Syndrome
PDK1	Phosphoinositide Dependent Kinase 1
PEG	PolyEthylene Glycol
PKC	Protein Kinase C
PLC	PhosphoLipase C
PI3K	Phosphoinositide-3-Kinase
PS	PhosphatidylSerine
PTEN	Phosphatase and TENsion homolog
PTP	Permeability Transition Pore
PTZ	PentyleneTetraZole
Rac1	Ras-related C3 botulinum toxin substrate 1
Raf	Rapidly accelerated fibrosarcoma
Ras	Rat sarcoma
RCC	Renal Cell Carcinoma
RGC	Retinal Ganglion Cells
ROS	Reactive Oxygen Species
ROS_i_	Intracellular Reactive Oxygen Species
ROS_m_	Mitochondrial Reactive Oxygen Species
RR	Ruthenium Red
RTX	ResiniferaToXin
RyR2	Ryanodine Receptor 2
SAEC	Small Airway Epithelial Cells
SCID-NOD	Severe Combined ImmunoDeficiency-NonObese Diabetic
SERCA	Sarco/Endoplasmic Reticulum Calcium ATPase
SGZ	SubGranular Zone
SMF	Static Magnetic Field
SNI	Sciatic Nerve Injury
SNP	Sodium NitroPrusside
SOC	Store-Operated Channel
SR	Sarcoplasmic Reticulum
Src	proto-oncogene tyrosine-protein kinase Src
SST	SomatoStaTin
sst_4_	somatostatin receptor 4
SVZ	SubVentricular Zone
T-ALL	T-cell Acute Lymphoblastic Leukemia
TG	Trigeminal Ganglia
TNF-α	Tumor Necrosis Factor α
TrkA	Tropomyosin receptor kinase A
TRPV1	Transient Receptor Potential Vanilloid 1
VDAC	Voltage Dependent Anion Channel
VGCC	Voltage Gated Calcium Channel
XBP1	X-box Binding Protein 1
