# Supplementary material for: Calcium Entry through TRPV1: A Potential Target for the Regulation of Proliferation and Apoptosis in Cancerous and Healthy Cells
Source: Int J Mol Sci. 2020 Jun 11;21(11):4177. doi: 10.3390/ijms21114177 (PMC7312732; doi:10.3390/ijms21114177)

Table S1. Proliferative effects of the exogenous TRPV1 agonists capsaicin and glycolic acid, the endogenous agonist AEA and its analogue, SKM-4-45-1, and the antagonists capsazepine and AMG9810. TRPV1-mediated proliferation is associated with Ca^{2+} influx, ATP release into the cytosol, and EGFR transactivation. Cancerous cell lines are highlighted in gray.

Item	Effect<br>Cell Line	Dose	Source
	Increases $[Ca^{2+}]_i$		
Glycolic Acid	In-vitro Reconstructed Skin Equivalent Model	100 mM; pH	[65]
	Causes ATP release		
Glycolic Acid	In-vitro Reconstructed Skin Equivalent Model	5 M; pH 2.4	[65]
	Upregulates (activates/phosphorylates) EGFR		
AMG9810	Skin tumors, DMBA-initiated SKH-1 mice	1 mg	[63]
AMG9810	N/TERT1	1 μM	[63]
Capsaicin	HCEC	10 μM	[62]
	Upregulates (activates/phosphorylates) Akt		
AMG9810	Skin tumors, DMBA-initiated SKH-1 mice	1 mg	[63]
AMG9810	Dorsal trunk skin, SKH-1 mice	1 mg	[63]
AMG9810	N/TERT 1	1 μM	[63]
Capsaicin	HCEC	10 μM	[62]
	Upregulates (phosphorylates) ERK 1/2		
Capsaicin	HCEC	10 μM	[62]
	Causes proliferation		
Glycolic Acid	In-vitro Reconstructed Skin Equivalent Model	1 M; pH 2.4	[65]
Capsaicin	In-vitro Reconstructed Skin Equivalent Model	10 μM	[65]
Capsaicin	ECFC	0.1 μM	[41]
AEA	ECFC	0.01-1 μM	[41]
SKM 4-45-1	ECFC	1 μM	[41]
Capsaicin	ASMC, Sprague-Dewley rats	1 μM	[35]
Capsaicin	ASMC, chronic asthmatic Sprague-Dewley	1 μM	[35]
Capsaicin	Eca109	--	[66]
Capsaicin	CF.41	0.0001-100 μM	[42]
Capsazepine	CF.41	0.0001-100 μM	[42]
AMG9810	Skin tumors, DMBA-initiated SKH-1 mice	1 mg	[63]
AMG9810	N/TERT1	1 μM	[63]
Capsaicin	HCEC	10 μM	[62]

Table S2. [Ca^{2+}]$_i$ elevation induced by various endogenous and exogenous TRPV1 agonists and activators. Modulation of the TRPV1 receptor by all of the listed substances and physiological conditions results in ion channel opening and Ca^{2+} influx. Cancerous cell lines are highlighted in gray.

Item	Effect<br>Cell Line	Dose Range	Source
	Increases [Ca^{2+}]$_i$		
Capsaicin	U87	50 μM	[49]
Capsaicin	U373	10-50 μM	[49]
Capsaicin	HCT116	50 μM	[48]
Capsaicin	HEK293T + TRPV1	--	[68]
Capsaicin	R2C Leydig	10 μM	[69]
Capsaicin	ASMC, chronic asthmatic Sprague-Dewley rats	1 μM	[35]
Capsaicin	Ishikawa	5 μM	[50]
Capsaicin	Neutrophils, adult PCOS humans	10 μM	[70]
Capsaicin	A2058	120 μM	[54]
Capsaicin	A375	120 μM	[54]
Capsaicin	HepG2	50 μM	[71]
Capsaicin	Synoviocytes, Wistar rat	10-100 μM	[39]
Capsaicin	NIH-3T3 + TRPV1	100 nM	[55]
Capsaicin	Primary breast epithelial cell, human	50 μM	[55]
Capsaicin	MCF-7	10-50 μM	[55]
Capsaicin	Osteoblasts, Wistar rat	1 μM	[72]
Capsaicin	SVZ NPCs, murine	1 μM	[37]
Capsaicin	SGZ NPCs, murine	1 μM	[37]
Capsaicin	Neuro2a	50 nM	[73]
Capsaicin	Sciatic nerve neurons, Wistar rat	10 μM	[65]
Capsaicin	DRG neurons, Wistar rat	10 μM	[65]
Capsaicin	HEK293T + TRPV1 + siControl	1 μM	[74]
Capsaicin	HEK293T + TRPV1 + siNCLX	1 μM	[74]
Capsaicin	DRG Neurons + siControl	1 μM	[74]
Capsaicin	DRG Neurons + siNCLX	1 μM	[74]
Capsaicin	DRG Neurons + siMCU	1 μM	[74]
Capsaicin	Hippocampal neurons, epileptic (PTZ) Wistar rat	100 μM	[75]
Capsaicin	DRG neurons, epileptic (PTZ) Wistar rat	100 μM	[75]
Capsaicin	DRG neurons, Wistar rat	100 μM	[75]
Capsaicin	MCF-7	0.00001-1000 μM	[76]
Capsaicin	LNCaP	0.005-5 μM	[46]
Capsaicin	PC-3	5 μM	[46]
AEA	Ishikawa	5 μM	[50]
AEA	MCF-7	0.00001-1000 μM	[76]
MET	LNCaP	~0.1-0.5 μM	[46]
MET	PC-3	0.1 μM	[46]
CBD	Ishikawa	5 μM	[50]
mNPC-CM	GL261	--	[43]
mNPC-CM	F98	--	[43]
mNPC-CM	U87	--	[43]
mNPC-CM	U373	--	[43]
mNPC-CM	Primary human glioblastoma	--	[43]
Arvanil	GL261	--	[43]

Hypoxia/Reoxygenation	H9C2	--	[38]
Wi-Fi	Hippocampal neurons, Wistar rat	2.45 GHz	[77]
Wi-Fi	Hippocampal neurons, epileptic (PTZ) Wistar rat	2.45 GHz	[77]
PTZ	Hippocampal neurons, Wistar rat	60 mg/kg	[77]
Hydrostatic Pressure	Primary culture retinal RGC, Sprague-Dewley rat	+70 mm Hg	[34]
Nonivamide	BEAS-2B + TRPV1 (over)	2.5 µM	[78]
SMF + Capsaicin	HepG2	0.5 T	[71]
Acidic Solution	Synoviocytes, Wistar rat	pH 5.5	[39]
Acidic Solution +	Synoviocytes, Wistar rat	pH 5.5	[39]
MRS1477	MCF-7	20 µM	[55]
MRS1477 + Capsaicin	MCF-7	20 µM	[55]
13-HODE	MCF-7	1 µM	[55]
EET	MCF-7	1 µM	[55]
SNP	Osteoblasts, Wistar rat	1 mM	[72]
ATP-P2Y2	Neuro2a	1-500 µM	[73]
SNI + Capsaicin	Sciatic nerve neurons, Wistar rat	10 µM	[65]
SNI + Capsaicin	DRG neurons, Wistar rat	10 µM	[65]
Residual Oil Fly Ash	SAEC	100 µg/mL	[79]
Residual Oil Fly Ash	TG neurons, TRPV1(+/+) mice	100 µg/mL	[79]
Mount St. Helen's Ash	NHBE	100 µg/mL	[79]
RTX	MCF-7	0.00001-1000 µM	[76]
RTX	LNCaP	0.01-1 µM	[46]
RTX	PC-3	0.01 µM	[46]

Table S3. Effects of the TRPV1 agonist capsaicin on the Fas/CD95 cytosolic pathway. ATM activates Fas/CD95, which co-clusters with TRPV1 to form a death signal complex. Procaspase 8 is cleaved from the zymogen form into caspase 8 by this complex, and cleaves BID into truncated BID. All listed cell lines are cancerous (highlighted in gray).

Item	Effect<br>Cell Line	Dose Range	Source
	Upregulates (activates/phosphorylates) ATM		
Capsaicin	RT4	100 μM	[52]
	Upregulates Fas/CD95 (protein)		
Capsaicin	RT4	100 μM	[52]
	Causes co-clustering of TRPV1 + Fas/CD95		
Capsaicin	RT4	100 μM	[52]
	Activates/upregulates caspase 8		
Capsaicin	786-O	100-300 μM	[45]
Capsaicin	RT4	100 μM	[52]
	Upregulates BID (mRNA)		
Capsaicin	RT4	100 μM	[52]
	Upregulates truncated BID (protein)		
Capsaicin	RT4	100 μM	[52]

Table S4. Mitochondrial pathway effects induced by TRPV1 agonists. Activation of TRPV1 modulates both $[Na^+]_m$ and $[Ca^{2+}]_m$. Initial cationic influx elevates the mitochondrial membrane potential, while passive and active transport of Ca^{2+} to the cytosol dissipates the potential and depolarizes the membrane. Membrane depolarization is the driving force behind ROS_m generation, and AIF and cytochrome c release. Cancerous cell lines are highlighted in gray.

Item	Effect<br>Cell Line	Dose Range	Source
	Causes mitochondrial Ca^{2+} influx/increases $[Ca^{2+}]_m$		
Capsaicin	U373	--	[49]
Capsaicin	HEK293T + TRPV1 + siControl	1 μM	[74]
Capsaicin	HEK293T + TRPV1 + siNCLX	1 μM	[74]
Capsaicin	DRG Neurons + siControl	1 μM	[74]
Capsaicin	DRG Neurons + siNCLX	1 μM	[74]
SNP	Osteoblasts, Wistar rat	1-4 mM	[72]
	Causes mitochondrial Na^+ influx/increases $[Na^+]_m$		
Capsaicin	HEK293T + TRPV1 + siControl	1 μM	[74]
Capsaicin	HEK293T + TRPV1 + siNCLX	1 μM	[74]
Capsaicin	DRG Neurons + siControl	1 μM	[74]
Capsaicin	DRG Neurons + siNCLX	1 μM	[74]
	Increases mitochondrial membrane potential		
Capsaicin	R2C Leydig	10 μM	[49]
	Dissipates/decreases mitochondrial membrane potential		
Capsaicin	U373	50 μM	[49]
Capsaicin	RT4	100 μM	[52]
Capsaicin	PC12	100-500 μM	[89]
Capsaicin	Synoviocytes, Wistar rat	100 μM	[39]
Hypoxia/Reoxygenation	H9C2	--	[38]
PTZ	Hippocampal neurons, Wistar rat	2.45 GHz	[77]
Wi-Fi	Hippocampal neurons, epileptic (PTZ) Wistar rat	2.45 GHz	[77]
Acidic Solution	Synoviocytes, Wistar rat	pH 5.5	[39]
Acidic Solution + Capsaicin	Synoviocytes, Wistar rat	pH 5.5	[39]
MRS1477	MCF7	2 μM	[55]
MRS1477 + Capsaicin	MCF7	2 μM	[55]
SNP	Osteoblasts, Wistar rat	1 mM	[72]
SNI + Capsaicin	Sciatic nerve neurons, Wistar rat	10 μM	[65]
SNI + Capsaicin	DRG neurons, Wistar rat	10 μM	[65]
	Causes PTP opening		
Capsaicin	U373	--	[49]
	Causes PS externalization		
Capsaicin	U373	50 μM	[49]
	Causes cytochrome c release		
Capsaicin	NPC-TW 039	300 μM	[87]
Capsaicin	RT4	100 μM	[52]
	Causes AIF release		
Capsaicin	NPC-TW 039	300 μM	[87]
	Increases ROS generation		
Capsaicin	786-O	100-300 μM	[45]
Capsaicin	R2C Leydig	10 μM	[49]

Capsaicin	ACHN	300 μM	[45]
Capsaicin	Meth A	100 μM	[47]
Capsaicin	CMS5	100 μM	[47]
Capsaicin	Synoviocytes, Wistar rat	100 μM	[39]
Hypoxia/Reoxygenation	H9C2	--	[38]
PTZ	Hippocampal neurons, Wistar rat	2.45 GHz	[77]
Wi-Fi	Hippocampal neurons, epileptic (PTZ) Wistar rat	2.45 GHz	[77]
Acidic Solution	Synoviocytes, Wistar rat	pH 5.5	[39]
Acidic Solution + Capsaicin	Synoviocytes, Wistar rat	pH 5.5	[39]
MRS1477	MCF7	2 μM	[55]
MRS1477 + Capsaicin	MCF7	2 μM	[55]
SNI + Capsaicin	Sciatic nerve neurons, Wistar rat	10 μM	[65]

Table S5. ER effects induced by endogenous and exogenous TRPV1 agonists. While TRPV1 receptors localized to the ER membrane are activated exclusively by endogenous agonists such as nonivamide, cell membrane TRPV1 receptors are subject to activation by all agonist types. Protein signaling by PERK, eiF2, IRE1 and ASK1 within the ER drive JNK activation and the upregulation of ATF4, AFT6, and XBP1. Efflux of Ca^{2+} contributes to dysregulation of $[Ca^{2+}]_i$. Cancerous cell lines are highlighted in gray.

Item	Effect<br>Cell Line	Dose Range	Source
	Activates (phosphorylates) p38/p38 MAPK		
Capsaicin	786-O	100-300 μM	[45]
Capsaicin	U373	50 μM	[49]
	Decreases ER Ca^{2+} content/causes Ca^{2+} efflux		
Capsaicin	PC12	50-500 μM	[89]
ATP-P2Y2	Neuro2a	100 μM	[73]
	Increases RyR2 Ca^{2+} channel mRNA expression		
Capsaicin	PC12	100-500 μM	[89]
	Decreases SERCA2 mRNA expression		
Capsaicin	PC12	50-500 μM	[89]
	Induces phosphorylated-eiF2 expression		
Capsaicin	GL261	--	[43]
mNPC-CM	GL261	--	[43]
Nonivamide	BEAS-2B	100-200 μM	[78]
	Induces/upregulates ATF4 mRNA expression		
Capsaicin	GL261	--	[43]
Capsaicin	PC12	50-500 μM	[89]
mNPC-CM	GL261	--	[43]
	Upregulates ATF4 protein expression		
Capsaicin	PC12	50-500 μM	[89]
	Upregulates ATF6 protein expression		
Capsaicin	NPC-TW 039	300 μM	[87]
	Increases GRP78 mRNA expression		
Nonivamide	BEAS-2B	100 μM	[78]
	Increases GRP78 protein expression		
Nonivamide	BEAS-2B	100 μM	[78]
Capsaicin	NPC-TW 039	300 μM	[87]
	Upregulates IRE1 protein expression		
Capsaicin	NPC-TW 039	300 μM	[87]
	Activates/phosphorylates JNK		
Capsaicin	786-O	100-300 μM	[45]
	Upregulates XBP1 mRNA expression		
Capsaicin	PC12	100-500 μM	[89]
	Upregulates XBP1 protein expression		
Capsaicin	PC12	100-500 μM	[89]

Table S6. Nuclear effects of endogenous and exogenous TRPV1 agonists. Cytosolic calcineurin and ATM contribute to upregulation of the p53 tumor suppressor gene through transcription factor activation and protein signaling. In turn, p53 upregulates the proapoptotic proteins p16, p21, and Bax. The nuclear component of ER stress occurs through a separate mechanism, in which the ATF4, ATF6, and XBP1 transcription factors upregulate the GADD153 transcription factor that downregulates Bcl-2. Cancerous cell lines are highlighted in gray.

Item	Effect<br>Cell Line	Dose Range	Source
	Activates calcineurin		
Capsaicin	HCT116	50 µM	[48]
Capsaicin	A2058	120 µM	[54]
Capsaicin	A375	120 µM	[54]
	Upregulates NFAT2		
Capsaicin	HCT116	50 µM	[48]
Capsaicin	A2058	120 µM	[54]
Capsaicin	A375	120 µM	[54]
	Downregulates p-NFAT2		
Capsaicin	A2058	120 µM	[54]
Capsaicin	A375	120 µM	[54]
	Upregulates c-myc		
Capsaicin	786-O	100-300 µM	[45]
	Induces E2F1 gene		
Capsaicin	RT4	100 µM	[52]
	Activates/upregulates p53/induces p53 gene		
Capsaicin	RT4	100 µM	[52]
Capsaicin	HCT116	50 µM	[48]
Capsaicin	A2058	120 µM	[54]
Capsaicin	A375	120 µM	[54]
Capsaicin	A2058 xenograft in SCID-NOD mice	1.8 mg	[54]
	Increases p53 acetylation		
Capsaicin	A2058	120 µM	[54]
Capsaicin	A375	120 µM	[54]
	Upregulates p21 mRNA expression		
Capsaicin	A2058	120 µM	[54]
Capsaicin	A375	120 µM	[54]
	Upregulates p21 protein expression		
Capsaicin	NPC-TW 039	300 µM	[87]
	Upregulates p16 protein expression		
Capsaicin	NPC-TW 039	300 µM	[87]
	Upregulates Bax		
Capsaicin	786-O	200-300 µM	[45]
Capsaicin	HCT116		[48]
Capsaicin	A2058	120 µM	[54]
Capsaicin	A375	120 µM	[54]
Capsaicin	PC12	100-500 µM	[89]
Capsaicin	HepG2	50 µM	[71]
Capsaicin	A172	50-100 µM	[100]
SMF + Capsaicin	HepG2	0.5 T	[71]

	ER Stress-Nuclear Activity		
	Increases GADD153 mRNA expression		
Nonivamide	BEAS-2B	100 μM	[78]
Nonivamide	BEAS-2B + TRPV1 (over)	1-2 μM	[78]
Nonivamide	NHBE	100 μM	[78]
Nonivamide	A549	100 μM	[78]
Resiniferatoxin	BEAS-2B + TRPV1 (over)	0.01 μM	[78]
Resiniferatoxin	BEAS-2B	7.5 μM	[78]
Resiniferatoxin	NHBE	7.5 μM	[78]
Resiniferatoxin	A549	7.5 μM	[78]
AEA	BEAS-2B + TRPV1 (over)	12.5 μM	[78]
AEA	BEAS-2B	25 μM	[78]
AEA	A549	25 μM	[78]
Capsaicin	PC12	50-500 μM	[89]
	Increases GADD153 protein expression		
Nonivamide	BEAS-2B	100-200 μM	[78]
Nonivamide	BEAS-2B + TRPV1 (over)	1-2 μM	[78]
Capsaicin	PC12	100-500 μM	[89]
	Downregulates Bcl-2		
Capsaicin	786-O	100-300 μM	[45]
Capsaicin	HCT116	50 μM	[48]
Capsaicin	A2058	120 μM	[54]
Capsaicin	A375	120 μM	[54]
Capsaicin	PC12	100-500 μM	[89]
Capsaicin	HepG2	50 μM	[71]
Capsaicin	A172	50-100 μM	[100]

Table S7. Effects of exogenous and endogenous TRPV1 agonists on caspase activity. Initial activation of caspase 9 is mediated by Bax protein, while p16 and p21 activate caspase 3. Once activated, caspase 9 also activates caspase 3.

Item	Effect<br>Cell Line	Dose Range	Source
	Activates/upregulates caspase 9		
Capsaicin	786-O	100-300 μM	[45]
Capsaicin	RT4	100 μM	[52]
Capsaicin	A172	50-100 μM	[100]
Capsaicin	MCF-7	100 μM	[55]
Capsaicin	DRG neurons, epileptic (PTZ) Wistar rat	100 μM	[75]
Capsaicin	Hippocampal neurons, epileptic (PTZ) Wistar rat	100 μM	[75]
Wi-Fi	Hippocampal neurons, epileptic (PTZ) Wistar rat	2.45 GHz	[77]
PTZ	Hippocampal neurons, Wistar rat	2.45 GHz	[77]
MRS1477	MCF-7	2 μM	[55]
MRS1477 + Capsaicin	MCF-7	2 μM	[55]
SNI + Capsaicin	Sciatic nerve neurons, Wistar rat	10 μM	[65]
SNI + Capsaicin	DRG neurons, Wistar rat	10 μM	[65]
	Activates/upregulates/cleaves caspase 3		
Capsaicin	786-O	100-300 μM	[45]
Capsaicin	U373	50 μM	[49]
Capsaicin	RT4	100 μM	[52]
Capsaicin	HCT116	50 μM	[48]
Capsaicin	R2C Leydig	10 μM	[69]
Capsaicin	Meth A	100 μM	[47]
Capsaicin	Hippocampal neurons, ICR mouse	10 μM	[101]
Capsaicin	Cortical neurons, ICR mouse	10 μM	[101]
Capsaicin	A2058	120 μM	[54]
Capsaicin	A375	120 μM	[54]
Capsaicin	HepG2	50 μM	[71]
Capsaicin	A172	50-100 μM	[100]
Capsaicin	MCF-7	100 μM	[55]
Capsaicin	DRG neurons, epileptic (PTZ) Wistar rat	100 μM	[75]
Capsaicin	Hippocampal neurons, epileptic (PTZ) Wistar rat	100 μM	[75]
Wi-Fi	Hippocampal neurons, epileptic (PTZ) Wistar rat	2.45 GHz	[77]
PTZ	Hippocampal neurons, Wistar rat	2.45 GHz	[77]
AEA	Ishikawa	5 μM	[73]
CBD	Ishikawa	5 μM	[73]
SMF + Capsaicin	HepG2	0.5 T	[78]
MRS1477	MCF-7	2 μM	[55]
MRS1477 + Capsaicin	MCF-7	2 μM	[55]
SNP	Osteoblasts, Wistar rat	1 mM	[72]
SNI	Sciatic nerve neurons, Wistar rat	N/A	[65]
SNI	DRG neurons, Wistar rat	N/A	[65]
SNI	Musculus piriformis, Wistar rat	N/A	[65]
SNI + Capsaicin	Sciatic nerve neurons, Wistar rat	10 μM	[65]
SNI + Capsaicin	DRG neurons, Wistar rat	10 μM	[65]

Table S8. Downstream pro-apoptotic effects of endogenous and exogenous TRPV1 agonists and antagonists. Caspases 9 and 3 cause DNA fragmentation and condensation in the nucleus. Degradation of DNA is a hallmark of apoptotic cell death. Cancerous cell lines are highlighted in gray.

Item	Effect<br>Cell Line	Dose Range	Source
	Causes nuclear/DNA fragmentation/condensation		
Capsaicin	U373	--	[49]
Capsaicin	786-O	--	[45]
Capsaicin	A172	100 μM	[100]
Capsaicin	Synoviocytes, Wistar rat	100 μM	[39]
AEA	Ishikawa	5 μM	[50]
CBD	Ishikawa	5 μM	[50]
2-AG	Ishikawa	5 μM	[50]
Acidic Solution	Synoviocytes, Wistar rat	pH 5.5	[39]
Acidic Solution + Capsaicin	Synoviocytes, Wistar rat	pH 5.5	[39]
	Causes apoptosis		
Capsaicin	786-O	100-300 μM	[45]
Capsaicin	HCT116	50 μM	[48]
Capsaicin	786-O xenograft (in vivo)	5 mg/kg in 100	[45]
Capsaicin	R2C Leydig	10 μM	[49]
Capsaicin	Meth A	100 μM	[47]
Capsaicin	Hippocampal neurons, ICR mouse	10 μM	[101]
Capsaicin	Cortical neurons, ICR mouse	10 μM	[101]
Capsaicin	Primary culture retinal RGC, Sprague-Dewley rat	1-100 μM	[35]
Capsaicin	A2058	120 μM	[54]
Capsaicin	A375	120 μM	[54]
Capsaicin	HepG2	50 μM	[71]
Capsaicin	DRG neurons, epileptic (PTZ) Wistar rat	100 μM	[75]
Capsaicin	Hippocampal neurons, epileptic (PTZ) Wistar rat	100 μM	[75]
Hypoxia/Reoxygenation	H9C2	--	[38]
Wi-Fi	Hippocampal neurons, epileptic (PTZ) Wistar rat	2.45 GHz	[77]
Hydrostatic Pressure	Primary culture retinal RGC, Sprague-Dewley rat	+70 mm Hg	[34]
SMF + Capsaicin	HepG2	0.5 T	[71]
MRS1477 + Capsaicin	MCF-7	2 μM	[55]
SNI + Capsaicin	Sciatic nerve neurons, Wistar rat	10 μM	[65]
Residual Oil Fly Ash	SAEC	100 μg/mL	[79]
Residual Oil Fly Ash	NHBE	100 μg/mL	[79]
Residual Oil Fly Ash	TG neurons, TRPV1(+/+) mice	100 μg/mL	[79]
Mount St. Helen's Ash	SAEC	100 μg/mL	[79]
Mount St. Helen's Ash	NHBE	100 μg/mL	[79]
Mount St. Helen's Ash	TG neurons, TRPV1(+/+) mice	100 μg/mL	[79]
	Decreases cell viability/Increases cell death		
Capsaicin	786-O	100-400 μM	[45]
Capsaicin	H9C2	1-100 μM	[38]
Capsaicin	Primary culture retinal RGC, Sprague-Dewley rat	1-100 μM	[34]
Capsaicin	A2058	120 μM	[54]
Capsaicin	A375	120 μM	[54]
Capsaicin	HepG2	50-200 μM	[71]
Capsaicin	A172	25-200 μM	[100]

Capsaicin	Synoviocytes, Wistar rat	10-100 μM	[39]
Capsaicin	MCF-7 + TRPV1 (over)	0.3-500 μM	[102]
Capsaicin	MCF-7	0.0001-100 μM	[76]
AEA	Ishikawa	5-25 μM	[50]
AEA	Hec50co	1-25 μM	[50]
CBD	Ishikawa	5-25 μM	[50]
CBD	Hec50co	25 μM	[50]
2-AG	Ishikawa	5-25 μM	[50]
2-AG	Hec50co	1-25 μM	[50]
RTX	MCF-7	0.0001-100 μM	[76]
Hypoxia/Reoxygenation	H9C2	--	[38]
Hypoxia/Reoxygenation	Primary cardiomyocytes, murine	--	[38]
Wi-Fi	Hippocampal neurons, epileptic (PTZ) Wistar rat	2.45 GHz	[77]
PTZ	Hippocampal neurons, Wistar rat	60 mg/kg	[77]
Nonivamide	BEAS-2B	2.5 μM	[78]
SMF + Capsaicin	HepG2	0.5 T	[71]
Acidic Solution	Synoviocytes, Wistar rat	pH 5.5	[39]
Acidic Solution + Capsaicin	Synoviocytes, Wistar rat	pH 5.5	[39]
MRS1477	MCF-7	2 μM	[55]
MRS1477 + Capsaicin	MCF-7	2 μM	[55]
SNP	Osteoblasts, Wistar rat	1 mM	[72]
SNI + Capsaicin	Sciatic nerve neurons, Wistar rat	10 μM	[65]
NADA	Cortical neurons, Wistar rat	3-30 μM	[36]
mNPC-CM	GL261	--	[43]
mNPC-CM	F98	--	[43]
mNPC-CM	U87	--	[43]
mNPC-CM	U373	--	[43]
mNPC-CM	Primary human glioblastoma	--	[43]
Arvanil	GL261	100 nM	[43]
Hydrostatic Pressure	Primary culture retinal RGC, Sprague-Dewley rat	+70 mm Hg	[34]
SMF + Capsaicin	HepG2	0.5 T	[71]
Capsazepine	MCF-7	0.0001-100 μM	[76]
I-RTX	MCF-7	0.0001-100 μM	[76]

Table S9. Effects of TRPV1 antagonists on transmembrane Ca^{2+} transport. Antagonists of the receptor reduce agonist-induced increases in TRPV1-mediated Ca^{2+} influx upon co-application. Alone, capsazepine attenuates passive Ca^{2+} influx through the TRPV1 channel. Cancerous cell lines are highlighted in gray.

Antagonist	With	Effect<br>Cell Line	Dose Range	Source
		Reduces agonist-induced Ca2+ influx		
Capsazepine	Capsaicin	U87	1 μM	[49]
Capsazepine	Capsaicin	U373	1 μM	[49]
Capsazepine	Capsaicin	ASMC, chronic asthmatic Sprague-Dewley	1 μM	[35]
Capsazepine	Capsaicin	Neutrophils, adult PCOS humans	10 μM	[70]
Capsazepine	Capsaicin	MCF-7	100 μM	
Capsazepine	Capsaicin	Osteoblasts, Wistar rat	10 μM	[72]
Capsazepine	mNPC-CM	GL261	1 μM	[43]
Capsazepine	mNPC-CM	F98	1 μM	[43]
Capsazepine	mNPC-CM	U87	1 μM	[43]
Capsazepine	mNPC-CM	U373	1 μM	[43]
Capsazepine	mNPC-CM	Primary human glioblastoma	1 μM	[43]
Capsazepine	Hypoxia/Reoxygenation	H9C2	1 μM	[38]
Capsazepine	Wi-Fi	Hippocampal neurons, Wistar rat	100 μM	[77]
Capsazepine	Wi-Fi	Hippocampal neurons, epileptic (PTZ)	100 μM	[77]
Capsazepine	Capsaicin	Synoviocytes, Wistar rat	2 μM	[39]
Capsazepine	Acidic Solution	Synoviocytes, Wistar rat	2 μM	[39]
Capsazepine	SNP	Osteoblasts, Wistar rat	10 μM	[72]
Capsazepine	Glycolic Acid	In-vitro Reconstructed Skin Equivalent	10 μM	[40]
Capsazepine	Capsaicin	Sciatic nerve neurons, Wistar rat	100 μM	[65]
Capsazepine	Capsaicin	DRG neurons, Wistar rat	100 μM	[65]
Capsazepine	SNI + Capsaicin	Sciatic nerve neurons, Wistar rat	100 μM	[65]
Capsazepine	SNI + Capsaicin	DRG neurons, Wistar rat	100 μM	[65]
Capsazepine	Capsaicin	DRG neurons, Wistar rat	100 μM	[75]
Capsazepine	Capsaicin	DRG neurons, epileptic (PTZ) Wistar rat	100 μM	[75]
Capsazepine	Residual Oil Fly Ash	SAEC	10 μM	[79]
Capsazepine	Mount St. Helen's Ash	NHBE	10 μM	[79]
Capsazepine	RTX	MCF-7	0.00001-1000	[76]
Caspazepine	Capsaicin	LNCaP	1 μM	[46]
Capsazepine	Capsaicin	PC-3	1 μM	[46]
Capsazepine	RTX	LNCaP	1 μM	[46]
Capsazepine	RTX	PC-3	1 μM	[46]
Capsazepine	MET	LNCaP	1 μM	[46]
Capsazepine	MET	PC-3	1 μM	[46]
I-RTX	Hydrostatic Pressure	Primary culture retinal RGC, Sprague-	10 nM	[34]
RR	Capsaicin	Synoviocytes, Wistar rat	10 μM	[39]
RR	Acidic Solution	Synoviocytes, Wistar rat	10 μM	[39]
RR	Capsaicin	Neuro2a	50 nM	[73]
RR	ATP-P2Y2	Neuro2a	50 nM	[73]
HP	Capsaicin	Sciatic nerve neurons, Wistar rat	30 mg/kg	[65]
HP	Capsaicin	DRG neurons, Wistar rat	30 mg/kg	[65]
HP	SNI + Capsaicin	Sciatic nerve neurons, Wistar rat	30 mg/kg	[65]

HP	SNI + Capsaicin	DRG neurons, Wistar rat	30 mg/kg	[65]
I-RTX	Capsaicin	Hippocampal neurons, epileptic (PTZ)	100 μg/kg	[75]
I-RTX	Capsaicin	Hippocampal neurons, Wistar rat	100 μg/kg	[75]
I-RTX	Capsaicin	DRG neurons, epileptic (PTZ) Wistar rat	100 μg/kg	[75]
I-RTX	Capsaicin	DRG neurons, Wistar rat	100 μg/kg	[75]
I-RTX	RTX	MCF-7	0.00001-1000	[76]
		Reduces Ca2+ influx below control level		
Capsazepine	Alone	ASMC, chronic asthmatic Sprague-Dewley	1 μM	[69]

Table S10. Effects of TRPV1 antagonists on mitochondrial dysfunction. Co-application of antagonists with agonists reduces Ca^{2+} movement into the mitochondrial matrix and stabilizes the mitochondrial membrane potential; downstream effects of mitochondrial membrane depolarization, such as ROS generation, are correspondingly diminished. Cancerous cell lines are highlighted in gray.

Antagonist	With	Effect<br>Cell Line	Dose Range	Source
		Reduces TRPV1 + Fas/CD95 co-clustering		
Capsazepine	Capsaicin	RT4	10 μM	[52]
		Reduces agonist-induced $[Ca^{2+}]_m$ increase		
Ruthenium Red	SNP	Osteoblasts, Wistar rat	10 μM	[72]
		Reduces agonist-induced MMP dissipation		
Capsazepine	Capsaicin	U373	1 μM	[49]
Capsazepine	Hypoxia/Reoxygenation	H9C2	1 μM	[38]
Capsazepine	Wi-Fi	Hippocampal neurons, epileptic (PTZ) Wistar	2.45 GHz	[77]
Capsazepine	Capsaicin	Synoviocytes, Wistar rat	2 μM	[39]
Capsazepine	Acidic Solution	Synoviocytes, Wistar rat	2 μM	[39]
Capsazepine	SNI + Capsaicin	Sciatic nerve neurons, Wistar rat	100 μM	[65]
Capsazepine	SNI + Capsaicin	DRG neurons, Wistar rat	100 μM	[65]
Ruthenium Red	SNP	Osteoblasts, Wistar rat	10 μM	[72]
HP	SNI + Capsaicin	Sciatic nerve neurons, Wistar rat	30 mg/kg	[65]
HP	SNI + Capsaicin	DRG neurons, Wistar rat	30 mg/kg	[65]
HP	SNI + Capsaicin	Sciatic nerve neurons, Wistar rat	30 mg/kg	[65]
		Increases MMP		
Capsazepine	Alone	R2C Leydig	100 μM	[69]
		Reduces agonist-induced PS externalization		
Capsazepine	Capsaicin	U373	1 μM	[49]
		Reduces agonist-induced ROS increase		
Capsazepine	Capsaicin	786-O	2 μM	[45]
Capsazepine	Hypoxia/Reoxygenation	H9C2	1 μM	[38]
Capsazepine	Capsaicin	Synoviocytes, Wistar rat	2 μM	[39]
Capsazepine	Acidic Solution	Synoviocytes, Wistar rat	2 μM	[39]
Capsazepine	MRS1477	MCF-7	100 μM	[55]
Capsazepine	MRS1477 + Capsaicin	MCF-7	100 μM	[55]
Capsazepine	SNI + Capsaicin	Sciatic nerve neurons, Wistar rat	100 μM	[65]

Table S11. Effects of TRPV1 antagonists on ER stress. Co-application of the antagonists capsazepine and LJO-328 with agonists stabilizes $[Ca^{2+}]_{ER}$ and attenuates agonist-induced protein signaling within the ER. Cancerous cell lines are highlighted in gray.

Antagonist	With	Effect<br>Cell Line	Dose Range	Source
		Reduces agonist-induced p38 MAPK		
Capsazepine	Capsaicin	U373	1 μM	[49]
		Reduces agonist-induced ER Ca^{2+} efflux		
LJO-328	Nonivamide	BEAS-2B + TRPV1 (over)	20 μM	[78]
		Reduces agonist-induced eiF2 upregulation		
LJO-328	Nonivamide	BEAS-2B	30 μM	[78]
		Reduces agonist-induced GRP78 upregulation		
LJO-328	Nonivamide	BEAS-2B	30 μM	[78]

′

Table S12. Effects of TRPV1 antagonists on nuclear activity. The receptor antagonists LJO-328 and IRTX diminish activation of the GADD153 protein, a nuclear transcription factor which enforces Bcl-2 downregulation and $[GSH]_i$ decrease which are downstream of ER stress. Cancerous cell lines are highlighted in gray.

Antagonist	With	Effect<br>Cell Line	Dose Range	Source
		Reduces agonist-induced GADD153		
LJO-328	Nonivamide	BEAS-2B	30-50 μM	[78]
LJO-328	Nonivamide	BEAS-2B + TRPV1 (over)	20 μM	[78]
LJO-328	Nonivamide	NHBE	50 μM	[78]
LJO-328	Nonivamide	A549	50 μM	[78]
I-RTX	Nonivamide	BEAS-2B + TRPV1 (over)	1 μM	[78]

Table S13. Effects of TRPV1 antagonists on caspase activity. A wide variety of receptor agonists, when applied in conjunction with agonists, reduce agonist-induced caspase 9 and 3 activation. Caspase activation is downstream of p53-upregulating nuclear activity. Cancerous cell lines are highlighted in gray.

Antagonist	With	Effect<br>Cell Line	Dose Range	Source
		Reduces agonist-induced caspase 9 activation		
Capsazepine	MRS1477 +	MCF-7	100 μM	[55]
Capsazepine	SNI + Capsaicin	Sciatic nerve neurons, Wistar rat	100 μM	[65]
Capsazepine	SNI + Capsaicin	DRG neurons, Wistar rat	100 μM	[65]
HP	SNI	Sciatic nerve neurons, Wistar rat	30 mg/kg	[65]
HP	SNI	DRG neurons, Wistar rat	30 mg/kg	[65]
HP	SNI	Skin cells, Wistar rat	30 mg/kg	[65]
HP	SNI	Musculus piriformis, Wistar rat	30 mg/kg	[65]
HP	SNI + Capsaicin	Sciatic nerve neurons, Wistar rat	30 mg/kg	[65]
HP	SNI + Capsaicin	DRG neurons, Wistar rat	30 mg/kg	[65]
I-RTX	Capsaicin	Hippocampal neurons, epileptic (PTZ) Wistar	100 μg/kg	[75]
I-RTX	Capsaicin	DRG neurons, epileptic (PTZ) Wistar rat	100 μg/kg	[75]
		Reduces agonist-induced caspase 3 activation		
Capsazepine	Capsaicin	U373	1 μM	[49]
Capsazepine	Wi-Fi	Hippocampal neurons, epileptic (PTZ) Wistar	2.45 GHz	[77]
Capsazepine	MRS1477	MCF-7	100 μM	[55]
Capsazepine	MRS1477 +	MCF-7	100 μM	[55]
Capsazepine	SNP	Osteoblasts, Wistar rat	10 μM	[72]
Capsazepine	SNI + Capsaicin	Sciatic nerve neurons, Wistar rat	100 μM	[65]
Capsazepine	SNI + Capsaicin	DRG neurons, Wistar rat	100 μM	[65]
Ruthenium Red	SNP	Osteoblasts, Wistar rat	30 μM	[72]
HP	SNI	Sciatic nerve neurons, Wistar rat	30 mg/kg	[65]
HP	SNI	DRG neurons, Wistar rat	30 mg/kg	[65]
HP	SNI	Skin cells, Wistar rat	30 mg/kg	[65]
HP	SNI	Musculus piriformis, Wistar rat	30 mg/kg	[65]
HP	SNI + Capsaicin	Sciatic nerve neurons, Wistar rat	30 mg/kg	[65]
HP	SNI + Capsaicin	DRG neurons, Wistar rat	30 mg/kg	[65]

Table S14. Effects of TRPV1 antagonists on agonist-induced apoptosis. Co-application of receptor antagonists with agonists downregulates apoptosis in vitro and in vivo. Cancerous cell lines are highlighted in gray.

Antagonist	With	Effect<br>Cell Line	Dose Range	Source
		Reduces agonist-induced DNA fragmentation		
Capsazepine	Capsaicin	U373	1 μM	[49]
		Reduces agonist-induced cell death/apoptosis		
Capsazepine	Capsaicin	786-O	2 μM	[45]
Capsazepine	mNPC-CM	GL261	1 μM	[43]
Capsazepine	mNPC-CM	F98	1 μM	[43]
Capsazepine	mNPC-CM	U87	1 μM	[43]
Capsazepine	mNPC-CM	U373	1 μM	[43]
Capsazepine	mNPC-CM	Primary human glioblastoma	1 μM	[43]
Capsazepine	Hypoxia/Reoxygenation	H9C2	1 μM	[38]
Capsazepine	Wi-Fi	Hippocampal neurons, epileptic (PTZ) Wistar	2.45 GHz	[77]
Capsazepine	Capsaicin	A2058	5 μM	[54]
Capsazepine	Capsaicin	A375	5 μM	[54]
Capsazepine	Capsaicin	Synoviocytes, Wistar rat	2 μM	[39]
Capsazepine	Acidic Solution	Synoviocytes, Wistar rat	2 μM	[39]
Capsazepine	MRS1477 + Capsaicin	MCF-7	100 μM	[55]
Capsazepine	SNI + Capsaicin	Sciatic nerve neurons, Wistar rat	100 μM	[65]
Capsazepine	Residual Oil Fly Ash	SAEC	10 μM	[79]
Capsazepine	Residual Oil Fly Ash	NHBE	10 μM	[79]
Capsazepine	Mount St. Helen's Ash	SAEC	10 μM	[79]
Capsazepine	Mount St. Helen's Ash	NHBE	10 μM	[79]
I-RTX	Hydrostatic Pressure	Primary culture retinal RGC, Sprague-Dewley	100 pM-100	[34]
HP	SNI + Capsaicin	Sciatic nerve neurons, Wistar rat	30 mg/kg	[65]
I-RTX	Capsaicin	Hippocampal neurons, epileptic (PTZ) Wistar	100 μg/kg	[75]
I-RTX	Capsaicin	Hippocampal neurons, Wistar rat	100 μg/kg	[75]
I-RTX	Capsaicin	DRG neurons, epileptic (PTZ) Wistar rat	100 μg/kg	[75]
I-RTX	Capsaicin	DRG neurons, Wistar rat	100 μg/kg	[75]
LJO-328	Nonivamide	BEAS-2B + TRPV1 (over)	20 μM	[78]
SB-705498	SMF + Capsaicin	HepG2	50 nM	[71]
HP	SNI + Capsaicin	Sciatic nerve neurons, Wistar rat	30 mg/kg	[65]

Figure S1. Activation of TRPV1 modulates pro- and anti-inflammatory signaling pathways. Agonists such as formaldehyde and particulate matter stimulate the release of substance P and CGRP, both pro-inflammatory neuropeptides, via TRPV1 signaling. Substance P and CGRP bind to their respective receptors and induce pro-inflammatory cytokine release and inflammation. Other agonists, particularly capsaicin, stimulate the release of the anti-inflammatory neuropeptide SST. SST binds to the sst_4 receptor, downregulates pro-inflammatory cytokines both directly and indirectly (*i.e.* through attenuation of substance P and CGRP release), and thus reduces inflammation.

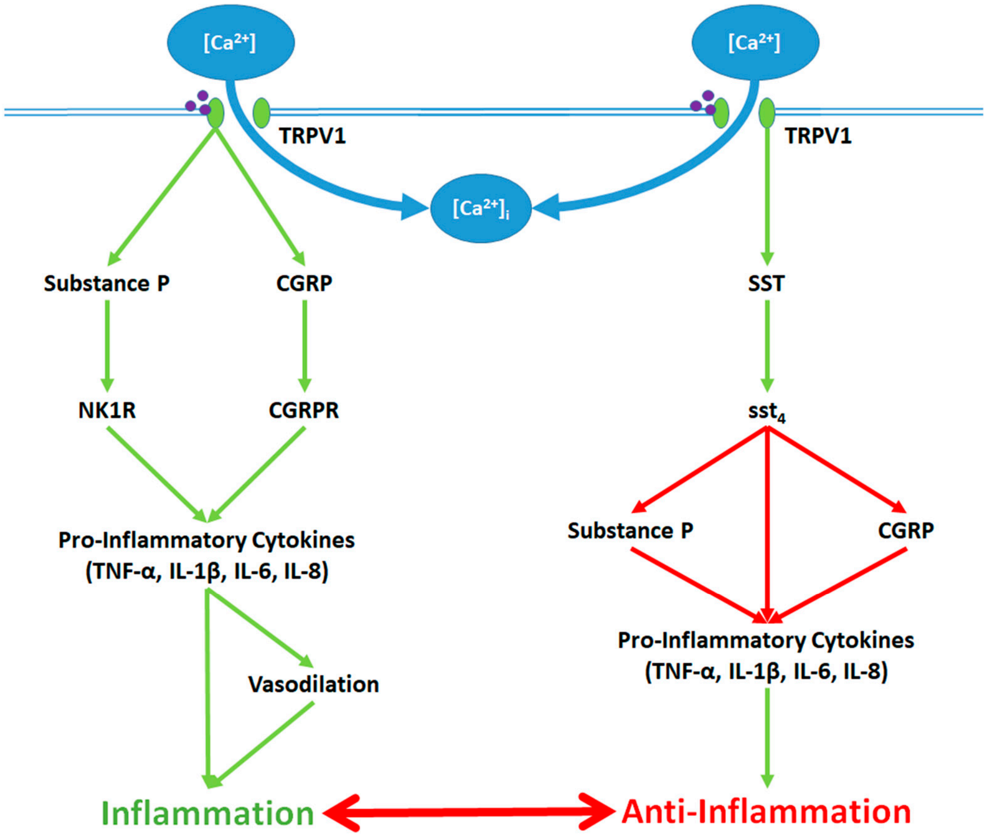

Supplement: Supplementary file 1 [file ijms-21-04177-s001.pdf]
